# Comparative analysis of the risks of hospitalisation and death associated with SARS-CoV-2 omicron (B.1.1.529) and delta (B.1.617.2) variants in England: a cohort study

**DOI:** 10.1016/S0140-6736(22)00462-7

**Published:** 2022-04-02

**Authors:** Tommy Nyberg, Neil M Ferguson, Sophie G Nash, Harriet H Webster, Seth Flaxman, Nick Andrews, Wes Hinsley, Jamie Lopez Bernal, Meaghan Kall, Samir Bhatt, Paula Blomquist, Asad Zaidi, Erik Volz, Nurin Abdul Aziz, Katie Harman, Sebastian Funk, Sam Abbott, COVID-19 Genomics UK (COG-UK) consortium, Tommy Nyberg, Neil M Ferguson, Sophie G Nash, Harriet H Webster, Seth Flaxman, Nick Andrews, Wes Hinsley, Jamie Lopez Bernal, Meaghan Kall, Samir Bhatt, Paula Blomquist, Asad Zaidi, Erik Volz, Nurin Abdul Aziz, Katie Harman, Sebastian Funk, Sam Abbott, Russell Hope, Andre Charlett, Meera Chand, Azra C Ghani, Shaun R Seaman, Gavin Dabrera, Daniela De Angelis, Anne M Presanis, Simon Thelwall, Russell Hope, Andre Charlett, Meera Chand, Azra C Ghani, Shaun R Seaman, Gavin Dabrera, Daniela De Angelis, Anne M Presanis, Simon Thelwall

**Affiliations:** a MRC Biostatistics Unit, University of Cambridge, Cambridge, UK; b NIHR Health Protection Research Unit for Modelling and Health Economics, MRC Centre for Global Infectious Disease Analysis, Jameel Institute, Imperial College London, London, UK; c NIHR Health Protection Research Unit for Respiratory Infections, Imperial College London, London, UK; d COVID-19 National Epidemiology Cell, UK Health Security Agency, London, UK; e COVID-19 Surveillance Cell, UK Health Security Agency, London, UK; f Outbreak Surveillance Team, UK Health Security Agency, London, UK; g COVID-19 Genomics Cell, UK Health Security Agency, London, UK; h Statistics, Modelling and Economics Department, UK Health Security Agency, London, UK; i Joint Modelling Team, UK Health Security Agency, London, UK; j Department of Computer Science, University of Oxford, Oxford, UK; k Centre for the Mathematical Modelling of Infectious Diseases, London School of Hygiene & Tropical Medicine, London, UK; l NIHR Health Protection Research Unit for Behavioural Science and Evaluation at the University of Bristol, University of the West of England, and University of Cambridge, Bristol, UK

## Abstract

**Background:**

The omicron variant (B.1.1.529) of SARS-CoV-2 has demonstrated partial vaccine escape and high transmissibility, with early studies indicating lower severity of infection than that of the delta variant (B.1.617.2). We aimed to better characterise omicron severity relative to delta by assessing the relative risk of hospital attendance, hospital admission, or death in a large national cohort.

**Methods:**

Individual-level data on laboratory-confirmed COVID-19 cases resident in England between Nov 29, 2021, and Jan 9, 2022, were linked to routine datasets on vaccination status, hospital attendance and admission, and mortality. The relative risk of hospital attendance or admission within 14 days, or death within 28 days after confirmed infection, was estimated using proportional hazards regression. Analyses were stratified by test date, 10-year age band, ethnicity, residential region, and vaccination status, and were further adjusted for sex, index of multiple deprivation decile, evidence of a previous infection, and year of age within each age band. A secondary analysis estimated variant-specific and vaccine-specific vaccine effectiveness and the intrinsic relative severity of omicron infection compared with delta (ie, the relative risk in unvaccinated cases).

**Findings:**

The adjusted hazard ratio (HR) of hospital attendance (not necessarily resulting in admission) with omicron compared with delta was 0·56 (95% CI 0·54–0·58); for hospital admission and death, HR estimates were 0·41 (0·39–0·43) and 0·31 (0·26–0·37), respectively. Omicron versus delta HR estimates varied with age for all endpoints examined. The adjusted HR for hospital admission was 1·10 (0·85–1·42) in those younger than 10 years, decreasing to 0·25 (0·21–0·30) in 60–69-year-olds, and then increasing to 0·47 (0·40–0·56) in those aged at least 80 years. For both variants, past infection gave some protection against death both in vaccinated (HR 0·47 [0·32–0·68]) and unvaccinated (0·18 [0·06–0·57]) cases. In vaccinated cases, past infection offered no additional protection against hospital admission beyond that provided by vaccination (HR 0·96 [0·88–1·04]); however, for unvaccinated cases, past infection gave moderate protection (HR 0·55 [0·48–0·63]). Omicron versus delta HR estimates were lower for hospital admission (0·30 [0·28–0·32]) in unvaccinated cases than the corresponding HR estimated for all cases in the primary analysis. Booster vaccination with an mRNA vaccine was highly protective against hospitalisation and death in omicron cases (HR for hospital admission 8–11 weeks post-booster *vs* unvaccinated: 0·22 [0·20–0·24]), with the protection afforded after a booster not being affected by the vaccine used for doses 1 and 2.

**Interpretation:**

The risk of severe outcomes following SARS-CoV-2 infection is substantially lower for omicron than for delta, with higher reductions for more severe endpoints and significant variation with age. Underlying the observed risks is a larger reduction in intrinsic severity (in unvaccinated individuals) counterbalanced by a reduction in vaccine effectiveness. Documented previous SARS-CoV-2 infection offered some protection against hospitalisation and high protection against death in unvaccinated individuals, but only offered additional protection in vaccinated individuals for the death endpoint. Booster vaccination with mRNA vaccines maintains over 70% protection against hospitalisation and death in breakthrough confirmed omicron infections.

**Funding:**

Medical Research Council, UK Research and Innovation, Department of Health and Social Care, National Institute for Health Research, Community Jameel, and Engineering and Physical Sciences Research Council.

## Introduction

During the COVID-19 pandemic, multiple variants of SARS-CoV-2 have emerged that have been found to vary in transmissibility and severity. The omicron (B.1.1.529) variant was first detected in a sample collected in Botswana on Nov 11, 2021 and first reported by South Africa on Nov 24, 2021.^1^, ^2^ Omicron was designated a variant of concern by WHO on Nov 26, 2021.^1^ To date, this variant has been identified in 133 countries and is now the most prevalent lineage globally, representing 85% of variant cases reported in late January, 2022.^3^ Delta (B.1.617.2) was the dominant variant in England between May and December, 2021. The first omicron case in England was reported on Nov 27, 2021, at a time when daily case numbers by specimen date had been 30 000–50 000 per day since July, 2021, and incidence of infection was estimated to be 40 000–80 000 per day over the same months.^4^ Since then, the number of confirmed omicron cases has rapidly increased in England; by the week commencing Jan 10, 2022, omicron cases represented more than 99% of all sequenced cases.^5^

**Research in context**

**Evidence before this study**

We aimed to identify all available evidence on the relative severity of omicron compared with other SARS-CoV-2 variants. On Jan 29, 2022, we searched PubMed with the query ((“B.1.1.529” OR “omicron” OR “VOC-21NOV-01”) AND (“SARS-CoV-2” OR “COVID-19” OR “severe acute respiratory syndrome coronavirus 2” OR “coronavirus disease 2019”)) AND (“severity” OR “hospitalisation” OR “hospitalization” OR “hospital” OR “emergency care” OR “mortality” OR “lethality” OR “death”), with no date or language restrictions. We further searched the medRxiv and SSRN preprint databases using combinations of the above search terms and included additional relevant literature from the reference lists of identified publications. This search identified three peer-reviewed publications and eight preprints. Comparing omicron with delta cases across all ages, published estimates of the reduction in the risk of hospitalisation or emergency department attendance ranged from 35% to 80%, with higher reductions in risk generally being reported for more severe outcomes, such as intensive care unit admission and death. A Norwegian cohort study reported age-stratified relative risk estimates and reported no differences with age for people younger than 75 years, but had insufficient power to disaggregate the younger than 30 years age group. Conversely, a Danish cohort study reported a relative risk of hospitalisation of 0·64 overall, but 1·59 in 0–19-year-olds, but again had insufficient power to disaggregate further. A US cohort study of children younger than 5 years reported relative risks of emergency department attendance and intensive care unit admission of 0·71 and 0·32, respectively.

**Added value of this study**

To date, this is the largest national study quantifying the risk of hospitalisation or death after infection with omicron compared with delta, based on individual-level data on 1 516 702 COVID-19 cases, of whom 1 067 859 were infected with the omicron variant. We provide age-specific estimates of the risk of hospitalisation and death for omicron relative to delta, and the disaggregation of the reduction in the risk of hospitalisation into estimates of the intrinsic severity reduction in unvaccinated cases and changes in vaccine-induced protection against hospitalisation in breakthrough cases. Furthermore, we estimate the additional protection provided by previous infection for unvaccinated and vaccinated individuals.

**Implications of all the available evidence**

The overall risk of severe outcomes for omicron infection is substantially lower than that for delta. However, this reduction in risk is age-specific and our results indicate that the risk of hospitalisation among children younger than 10 years does not significantly differ between omicron and delta. The reduction in the risk of hospitalisation observed at the population level is composed of a larger reduction in intrinsic severity coupled with a moderate reduction in the protection afforded by vaccination against hospitalisation.

A number of studies have indicated that the clinical severity of infection is lower for omicron than for delta.^6^, ^7^, ^8^, ^9^, ^10^, ^11^, ^12^, ^13^, ^14^, ^15^, ^16^, ^17^ Vaccine effectiveness estimates show reduced protection against symptomatic infection for omicron compared with that of delta, with this protection being low after the primary course, but moderate against symptomatic infection and high against hospitalisation after a booster dose.^18^ In England, such estimates have been obtained in a context of high vaccination coverage. By Jan 9, 2022, more than 95% of people in England aged over 70 years had received one vaccine dose, more than 93% had received two vaccine doses, and more than 90% had received three vaccine doses.^19^ Coverage, notably of boosters, was lower in younger age groups, with 81%, 52%, and 43% having received three doses in the 55–59 years, 40–44 years, and 30–34 years age groups, respectively.^19^

However, a detailed understanding is needed of how reductions in both severity and immunity have shaped observed patterns of hospitalisations and deaths in the omicron wave, and there has been scarce characterisation of age variation in the severity of omicron infection to date. To inform the public health response, we aimed to assess the relative risks of hospitalisation and death by age for infection with omicron versus delta. We also sought to estimate how immunity from both vaccination and past infection modifies disease severity in breakthrough cases.

## Methods

### Data sources

COVID-19 is a notifiable disease and data for all positive cases in England are reported to the UK Health Security Agency (UKHSA), which maintains a definitive line list of all individuals who have had confirmed SARS-CoV-2 infection.^20^ UKHSA also maintains separate line lists of COVID-19-associated deaths, reinfection episodes, S-gene target failure data, and sequencing and genotyping test results. The UK sequencing and genotyping strategy has been previously described.^21^, ^22^ Briefly, this strategy includes geographically weighted, population-level sampling of community cases, supplemented by targeted sampling of recent international travellers, hospitalised cases, and hospital staff. Over the study period, 6–20% of cases were sequenced, and 16–35% of cases were either sequenced or genotyped. S-gene information was available from the three largest PCR-testing laboratories using TaqPath assays, covering 47% of cases identified from community testing during the inclusion period.^23^ SARS-CoV-2 vaccinations are recorded in the National Immunisation Management Service.^24^ Hospital attendance data are recorded in the Emergency Care Data Set^25^ and Secondary Uses Service^26^ datasets. We linked these datasets by National Health Service (NHS) number, a unique individual identifier.

### Study design and participants

In this retrospective cohort study, we included individuals resident in England with laboratory-confirmed SARS-CoV-2 infection with a specimen date in the 6 weeks between Nov 29, 2021, and Jan 9, 2022. Cases were retrospectively followed up until Jan 24, 2022, the date of data extraction. Cases were included (appendix p 2) if their specimen was whole-genome sequencing-confirmed omicron or delta SARS-CoV-2; genotyping-confirmed omicron or delta SARS-CoV-2; or, if no known sequencing or genotyping-confirmed variant information was available, S-gene negative (omicron) or S-gene positive (delta) SARS-CoV-2. S-gene data were only used for test dates up to and including Dec 30, 2021, since increasing incidence of the S-gene positive BA.2 lineage of omicron reduced the positive predictive value of S-gene positivity for delta after that date (appendix p 4). Reinfections were defined as two positive tests in the same individual taken more than 90 days apart. If there were multiple positive tests within 90 days of each other for the same individual, these were attributed to the same infection episode and the earliest test date in that episode was used.

Cases were excluded if the NHS number recorded was missing or invalid (since such cases could not be linked to hospitalisation or vaccination records); information was missing for any adjustment variables; there were more than 14 days between the date of the first positive test and the date of the test which led to the variant being identified (via sequencing, genotyping, or S-gene positivity); or the specimen date was after an individual had died. We also excluded a small number of cases in individuals who had received (1) a vaccine other than Oxford–AstraZeneca, Pfizer–BioNTech, or Moderna or more than three doses of vaccine; (2) a third dose of vaccine that was not Pfizer or Moderna; or (3) a third dose of vaccine less than 80 days after the second dose.

Three hospitalisation outcomes, of differing severity level, were examined: hospital admissions, hospital attendances (including admissions), and hospital attendances (including admissions and diagnoses during hospital stay). Specifically, the hospital admissions outcome was defined as hospital admissions occurring 0–14 days after the first positive specimen date of the most recent infection episode, where either length of stay in hospital was 1 or more days; the Emergency Care Data Set discharge field recorded a patient as admitted or transferred; or the patient died in hospital on the same day as hospital attendance. Hospital attendances (including admissions) was defined as any hospital attendance, including admissions and attendances at accident and emergency departments, 0–14 days after the first specimen date of the most recent infection episode. Hospital attendances (including admissions and diagnoses during hospital stay) was defined in the same way as hospital attendances (including admissions), but additionally included cases with a first specimen date occurring during their hospital stay (hospital-onset cases^27^), to approximately match the definition used in NHS COVID-19 hospitalisation statistics.^28^ The mortality outcome was defined as death occurring 0–28 days after the first positive specimen date of the most recent infection episode, again matching the definition used in routine UK government reporting.

The surveillance activities within which this study was conducted are part of UKHSA's responsibility to monitor COVID-19 during the current pandemic. UKHSA has legal permission, provided by Regulation 3 of The Health Service (Control of Patient Information) Regulations 2002 to process confidential patient information under Sections 3(i) a–c, 3(i) d(i and ii), and 3(iii) as part of its outbreak response activities. This study falls within the research activities approved by the UKHSA's Research Ethics and Governance of Public Health Practice Group. Data were shared with the investigators as part of the UK's emergency response to the COVID-19 pandemic, via the scientific pandemic influenza group on modelling (SPI-M) subcommittee of the UK Scientific Advisory Group for Emergencies. Ethics permission was sought for analyses of these data via Imperial College London's standard ethical review processes and the study was approved by the College's Research Governance and Integrity Team.

### Statistical analysis

Stratified Cox proportional hazards regression was used to estimate hazard ratios (HRs) for the hospitalisation and mortality outcomes. For the hospitalisation outcomes, cases were followed up from the specimen date until hospitalisation, or censored at the earliest of: death, date of data extraction, and 14 days after their specimen date. For the death outcome, cases were followed up for 28 days. If an outcome occurred on or before the specimen date, the follow-up time was taken to be 0·5 days.

The model used for the primary analysis was stratified by date of specimen, NHS region of residence of the case, 10-year age band, ethnicity group, and vaccination status (defined as vaccine of the primary series [Oxford–AstraZeneca *vs* Pfizer–BioNTech or Moderna] and the number of doses received); and further included regression adjustments for sex, index of multiple deprivation (a measure of socioeconomic deprivation for the local area), year of age within each age band, and an interaction term between previous infection status and any history of vaccination (to allow the effect of previous infection to vary by vaccination status).

The secondary analysis differed from the primary analysis in removing vaccination status from the stratification and instead simultaneously estimating omicron:delta HRs for unvaccinated individuals, variant-specific HRs for different vaccination strata compared with unvaccinated cases, and vaccination status-specific HRs for cases with previous infection compared with those without previous infection.

In sensitivity analyses, we examined the effects of finer age stratification (appendix p 10); restriction to the subgroup of unvaccinated cases (appendix p 11); interaction of past infection status with variant (appendix p 12); alternative variant classifications (appendix p 13); alternative adjustment or stratification strategies (or both; appendix pp 14–15); alternative definitions of hospitalisation endpoints (appendix p 16); epidemic phase bias^29^ (appendix p 17); and adjusting for under-ascertainment of past infection status (appendix pp 18–21).

Data were prepared and statistical analyses were carried out using R (version 4.1.12; R Foundation for Statistical Computing, Vienna, Austria).

### Role of the funding source

The funders of the study had no role in study design, data collection, data analysis, data interpretation, or writing of the report.

## Results

**Figure 1:**
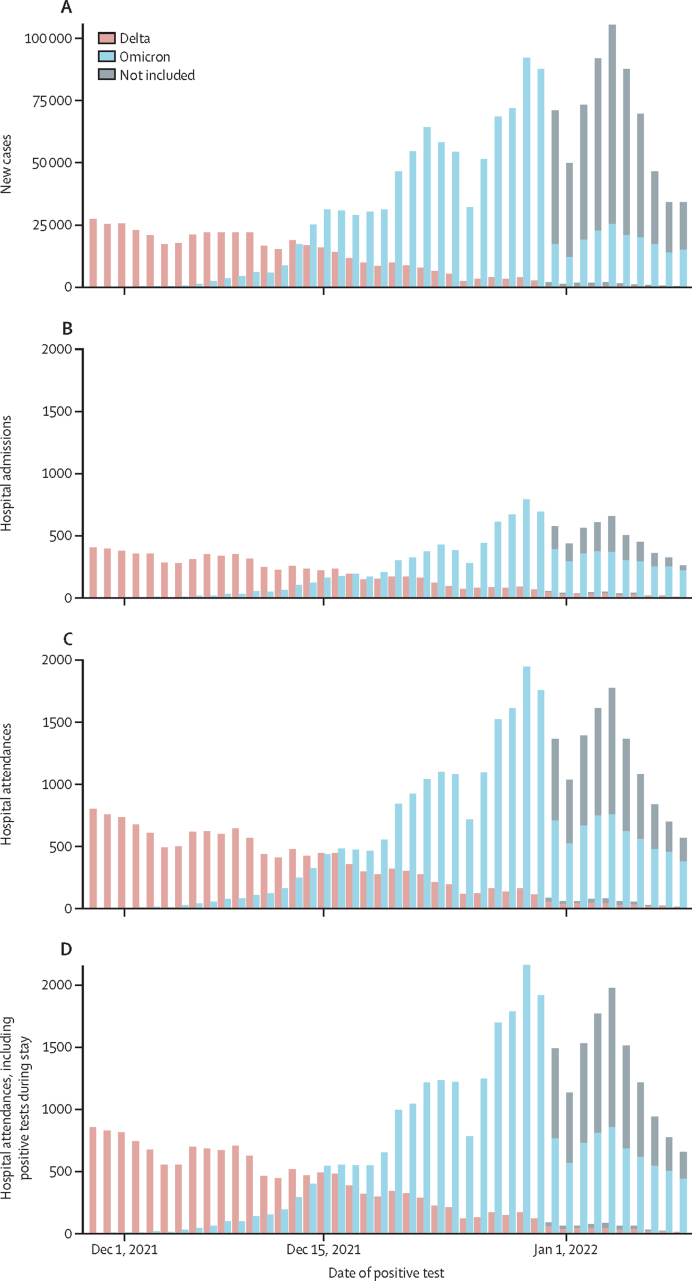
Cases, hospital admissions, and hospital attendances in those with delta and omicron SARS-CoV-2 variants, between Nov 29, 2021, and Jan 9, 2022 Plots show number of cases (A), number of hospital admissions (B), number of hospital attendances, including admissions (C), and number of hospital attendances, including admissions and diagnoses during hospital stay (D), by variant and date of positive test. We included cases whose positive specimen had been classified as delta or omicron based on (1) whole-genome sequencing or genotyping or (2) for positive tests until Dec 30, 2021, cases whose positive specimen was assessed for S-gene target failure. For illustration purposes, the figure shows the number of cases with S-gene information (in grey) from Dec 31, 2021, but these cases were not included in the analysis.

Between Nov 29, 2021, and Jan 9, 2022, 4 135 347 COVID-19 cases were detected in England, of which 1 516 702 (37%) had available variant classification data and met the criteria to be included in the analysis (figure 1; appendix p 2). These comprised 448 843 delta and 1 067 859 omicron cases (appendix p 6), with 5983 (1·3%) delta cases and 102 957 (9·6%) omicron cases (appendix p 6) being reinfections of people with documented earlier infection 90 or more days before their latest confirmed infection. The appendix (p 6) shows the distribution of omicron and delta cases by age, sex, ethnicity, residential region, index of multiple deprivation, week of specimen date, vaccination category, and previous infection status. The proportion of cases with omicron increased steadily each week during December, 2021. Individuals with omicron were more likely than delta cases to be from a Black ethnic group, or to live in London or the northwest of England. The patterns of number of cases detected and hospitalisations were similar between the included cases and all cases during the inclusion period (figure 1, appendix p 4). Compared with cases included in the analysis, cases in individuals who did not fulfil the inclusion criteria had similar characteristics but were slightly more likely to reside in London or the east of England and to be from non-White ethnic groups. As expected, a high proportion of cases were not included after December 30, corresponding to the time when sequencing coverage decreased and S-gene target failure information was not used due to the increase in BA.2 cases (appendix p 6).

**Figure 2:**
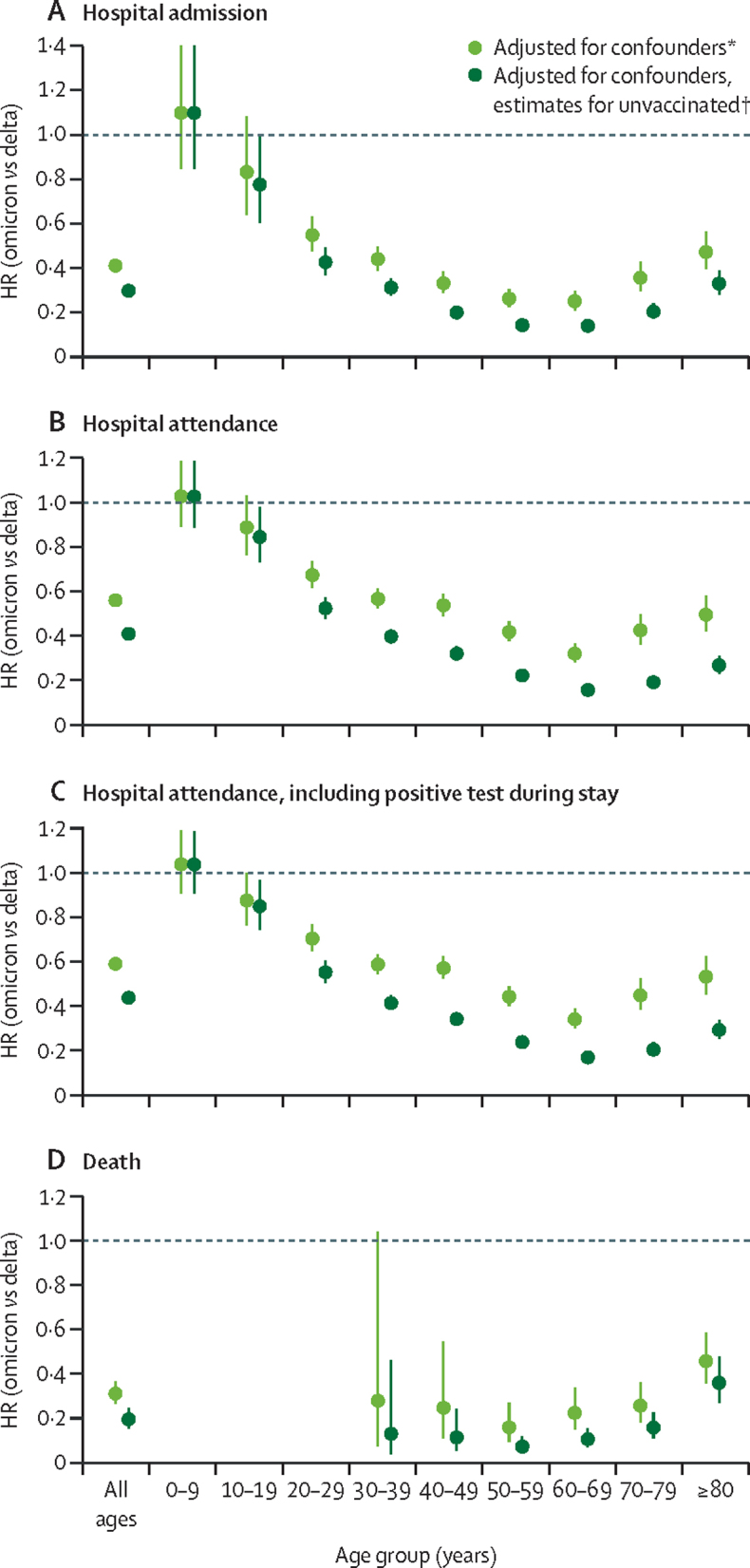
Risk of hospitalisation and mortality for COVID-19 cases with omicron compared with delta, overall and by age group Plots show omicron versus delta adjusted HRs for the four endpoints: hospital admission (A), hospital attendance, including admission (B), hospital attendance, including admission and diagnosis during hospital stay (C), and death (D). Error bars are 95% CIs. HRs of death were not estimated for individuals younger than 30 years due to small numbers. Counts and HR estimates (unadjusted and adjusted) are given in the appendix (p 7). HR=hazard ratio.*HRs from the primary analysis, adjusted (by stratification) for date of specimen, National Health Service region of residence of the case, 10-year age band, ethnicity group, and vaccination status, and adjusted (by regression) for sex, index of multiple deprivation, year of age within each age band, and an interaction term between previous infection status and any history of vaccination. †HRs from the secondary analysis, for the unvaccinated group only (intrinsic severity), adjusted for the same confounders, but with vaccination status not used as a stratification variable, but instead used to simultaneously estimate the omicron versus delta HRs for unvaccinated individuals, variant-specific HRs for each vaccination category compared with unvaccinated (figure 3), and vaccination status-specific HRs for cases with a previous infection compared with those without.

Figure 2 presents estimates of the adjusted HRs for hospitalisation and mortality endpoints comparing omicron with delta, across all ages and stratifying by 10-year age group, from both the primary and the secondary analyses (appendix pp 7–9). Unadjusted HRs (appendix p 7) give a biased picture of relative severity due to differences in the distribution of omicron and delta cases over time, by age (even within age bands), by ethnicity, and by residential region. The adjustment for single year of age within age band is particularly important for the youngest band (<10 years), where differences in the detailed age distribution of omicron and delta cases (appendix p 4) can bias estimates otherwise. For the least severe and least COVID-19-specific endpoint examined, hospital attendances (including admissions and diagnoses during hospital stay), the adjusted HR estimate (0·59, 95% CI 0·57–0·61) indicates a lower risk of hospitalisation with omicron versus delta, averaging over all age groups and vaccination strata. The adjusted HR estimate for attendances, including admissions but not diagnoses during hospital stay, is similar: 0·56 (0·54–0·58). A greater reduction is estimated for the most severe and specific hospital endpoint, hospital admission up to 14 days after a positive test (adjusted HR 0·41, 0·39–0·43), with the greatest reduction estimated in the risk of death within 28 days of a positive test (adjusted HR 0·31, 0·26–0·37).

We found considerable variation in the severity of omicron relative to delta cases with age for all endpoints examined (figure 2). The HRs for hospitalisation did not differ between the two variants in individuals younger than 10 years (adjusted HR for admission 1·10 [95% CI 0·85–1·42]); only small (and sometimes non-significant) reductions in the risk of hospitalisation were seen in 10–19-year-olds, with increasingly large reductions seen with age in 20–69-year-olds (adjusted HR for admission in 60–69-year-olds 0·25 [0·21–0·30]). Although HR estimates increased again with age in those aged at least 70 years (adjusted HR for admission in those aged at least 80 years 0·47 [0·40–0·56]), they remained substantially below 1. Similar trends were seen for the mortality endpoint in adults over 30 years, but we had insufficient power to examine this endpoint in the younger age groups. In a secondary analysis (appendix p 10), we disaggregated the younger than 10 years age group into three bands (<1 year, 1–4 years, and 5–9 years). The results are suggestive of a higher relative risk in those younger than 1 year than in those aged 1–4 years or 5–9 years, but these differences were not statistically significant.

**Figure 3:**
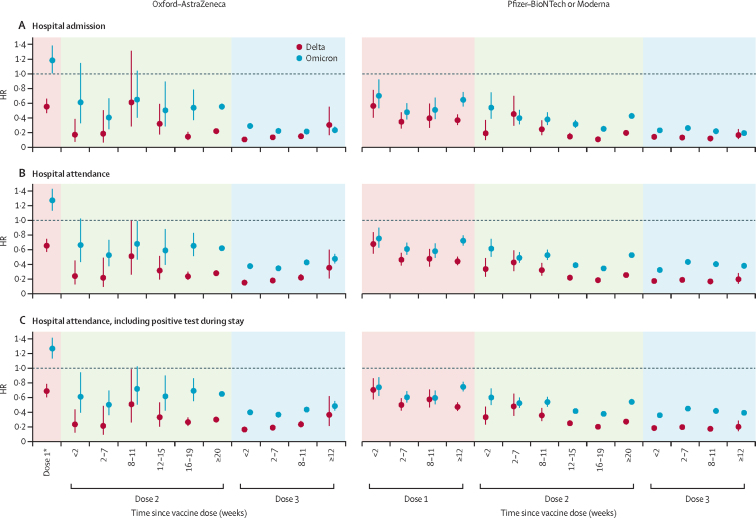
Estimated HRs for vaccination categories, secondary analysis Variant-specific HRs of hospital admission (A), any hospital attendance, including admission (B), or any hospital attendance, including admission or positive test during hospital stay (C), by type of vaccine used for doses 1 and 2, number of vaccine doses, and time since last dose, relative to unvaccinated cases. These HRs can be interpreted as 1 – vaccine effectiveness at preventing hospitalisation conditional upon diagnosed infection. Booster doses were Pfizer–BioNTech or Moderna (not disaggregated). HR=hazard ratio. *Due to small numbers, all cases who had received a single dose of the Oxford–AstraZeneca vaccine were grouped together and not separated by time since vaccine dose.

There was a high level of immunity in the population of England by the time omicron emerged, both from vaccination and previous infection. It is, therefore, informative to disaggregate the HRs for hospitalisation and death into estimates of changes in intrinsic viral severity applying to unvaccinated cases (figure 2, secondary analysis), and changes in the protection vaccines afford against severe outcomes in breakthrough infections. We estimated a larger reduction (comparing omicron with delta) in the risk of hospitalisation and death in unvaccinated cases than for all cases (figure 2, appendix pp 8–9). Figure 3 presents estimates of variant-specific HRs of each hospitalisation endpoint for different vaccination strata compared with unvaccinated cases (appendix pp 8–9). The relative risk of hospitalisation or death in vaccinated cases compared with unvaccinated cases was lower for delta cases than for omicron cases (figure 3, appendix pp 8–9). These estimates indicate that the overall observed reductions in hospitalisation and mortality risk understate the intrinsic reduction in the risk of severe infection outcomes associated with the delta to omicron transition, due to those reductions being partially counteracted by reductions in vaccine effectiveness. The largest reductions in vaccine effectiveness against hospitalisation in breakthrough cases were seen for people who had not received a booster dose, particularly for those who had received the Oxford–AstraZeneca vaccine for their primary vaccination series (figure 3). However, relative protection (*vs* unvaccinated) against hospital admission with omicron in breakthrough cases remained above 70% (HR <0·3) for all vaccination categories that included a booster dose (adjusted HR for hospital admission 8–11 weeks post-booster *vs* unvaccinated 0·22 [95% CI 0·20–0·24]; appendix pp 8–9). Documented past infection was found to protect unvaccinated cases against hospitalisation (appendix pp 8–9), with HR estimates of 0·55 (95% CI 0·48–0·63) for hospital admission and 0·18 (0·06–0·57) for death. These estimates imply a similar level of protection against hospitalisation as that provided by two doses of the Oxford–AstraZeneca vaccine or one dose of the Pfizer or Moderna vaccines (appendix pp 8–9). In vaccinated cases, past infection did not provide additional protection against hospitalisation (HR for admission 0·96, 0·88–1·04) beyond that afforded by vaccination, but did provide additional protection against death (HR 0·47, 0·32–0·68). We found no evidence that the protection against hospitalisation or death afforded by documented past infection differed significantly between omicron and delta cases (appendix p 12).

Although the HR estimates for unvaccinated cases in figure 2 were derived from a model fitted to all cases, adjusted HR estimates were similar for a model fitted to the subgroup of unvaccinated cases only (appendix p 11). A sensitivity analysis using imputation to account for under-ascertainment of past infection (appendix pp 18–21) gave, as expected,^8^ slightly higher estimates of omicron:delta HRs than our secondary analysis did (appendix pp 8–9), but slightly lower estimates of the HRs for vaccinated cases versus unvaccinated for omicron, and lower estimates of the HRs for reinfections versus first infections. Other sensitivity analyses (appendix p 13) indicated that HR estimates did not vary significantly if only Pillar 2 (community testing) cases were included in the analysis, but were slightly closer to 1 than in the primary analysis if only sequencing data were used to determine variant status, and slightly further from 1 than the primary analysis if sequencing and genotyping (but not S-gene target failure data) were used. Estimates were relatively robust to alternative choices of adjustment or stratification (appendix pp 14–15) and to precise inclusion criteria for hospital endpoints (appendix p 16). As expected, exploratory analysis of the potential effects of epidemic phase bias^29^ indicated that the primary analysis might overestimate the omicron versus delta HR if the delay from infection to test is substantially shorter in hospitalised cases than in non-hospitalised cases (appendix p 17).

## Discussion

Since mid-December, 2021, most new SARS-CoV-2 cases in England have been caused by the omicron variant. Our results suggest that confirmed omicron cases had a 59% lower risk of hospital admission, a 44% lower risk of any hospital attendance, and a 69% lower risk of death than that of confirmed delta cases. We found strong evidence of age-dependence in the magnitude of this risk reduction. In those over 20 years of age, we estimate a significant reduction in the risk of hospitalisation for omicron compared with delta. In cases aged 50 years and above, the estimated reduction in the risk of hospitalisation is 50–75%, depending on the endpoint examined. The magnitude of severity reduction is lower for those older than 80 years, but still over 50% for most endpoints. In individuals aged 0–9 years with a confirmed infection, for whom the risk of disease sufficiently severe to result in death is very low, we estimate that the risk of hospitalisation from omicron infection is not significantly different from that of delta infection. Dividing the 0–9 years age group into three bands (<1 year, 1–4 years, and 5–9 years) indicated no significant differences in the omicron:delta HR with age in that age group, although statistical power was more restricted for this finer disaggregation of age. The absence of a difference in the risk of hospitalisation for young children between omicron and delta might be explained by a different clinical presentation of omicron infection in children. Laboratory studies have shown that omicron replicates more in upper airway cells and less in the lungs.^30^, ^31^ Children with omicron infection may, therefore, be more likely than those with delta infection to present with fever and upper respiratory symptoms that would trigger clinical pathways for admission, for example to rule out sepsis.^32^, ^33^ Thus, the absence of severity attenuation in children might reflect a lower clinical threshold for hospital admission for young children due to symptoms common to omicron infection, rather than more severe disease.^6^, ^33^ However, further analyses are required to investigate COVID-19 illness in children and reasons for admission.

We report that pre-existing immunity, both from vaccination and past infection, substantially reduces the risk of hospitalisation. In a secondary analysis, we estimated both variant-specific vaccine effectiveness and age-specific risks of hospitalisation in unvaccinated cases. We estimated lower severity for omicron versus delta compared with the primary analysis, where vaccination status was included in the stratification. This finding is explained by the lower estimates of protection afforded by vaccination against hospitalisation in breakthrough cases for the omicron variant compared with the delta variant. Hence, underlying the observed risks for hospitalisation and death in our primary analysis is a larger reduction in intrinsic severity (ie, for unvaccinated cases) counterbalanced by a reduction in vaccine effectiveness against omicron compared with delta. However, mRNA booster vaccination was still found to be highly protective against hospitalisation and death in omicron breakthrough cases, in line with studies solely examining vaccine effectiveness.^18^ In unvaccinated cases, documented past infection provides moderate protection against hospitalisation and higher protection against death. In vaccinated cases, past infection offered no additional protection against hospitalisation over vaccination alone, but did offer moderate additional protection against death.^18^ An imputation-based sensitivity analysis to examine the effect of under-ascertainment of past infections gave slightly higher estimates of the severity of omicron relative to delta, together with estimates indicating a larger protective effect of past infection against all endpoints for unvaccinated individuals, and against hospital admission and death in vaccinated individuals.^8^

To our knowledge, this is the largest study to date to report on the relative hospitalisation and mortality risks for cases with the omicron variant compared with delta. Strengths of the study include the use of a nationwide cohort, covering 37% of all COVID-19 cases in England during the inclusion period. Our results support those of a number of other studies which have reported that omicron has substantially reduced overall severity compared with delta.^6^, ^7^, ^8^, ^9^, ^10^, ^11^, ^12^, ^13^, ^14^, ^15^, ^16^, ^17^ However, relatively few studies have examined how the reduction in severity might vary with age. A US study^12^ compared 3-day follow-up outcomes in individuals younger than 5 years in the previous delta epidemic wave with those in the ongoing omicron epidemic and found a relative risk of emergency department attendance of 0·71 (95% CI 0·66–0·75) and a relative risk of admission of 0·33 (0·26–0·43). A second study involved a contemporaneous comparison of omicron and delta (distinguished via S-gene target failure) cases in southern California^10^ and estimated unadjusted odds ratios for hospitalisation in 0–17-year-olds of 0·70 (95% CI 0·12–3·97) and 0·94 (0·26–3·42) using denominators of only outpatient tests and both inpatient and outpatient tests, respectively. A Danish study^11^ estimated a relative risk of hospitalisation of 1·59 (1·09–2·32) in 0–19-year-olds, although this estimate was based on only 31 hospitalisations in that age group. Last, a Norwegian cohort study^13^ found no significant differences by age in the relative risk of hospitalisation for people younger than 75 years, with a point estimate for individuals younger than 30 years of 0·24 (95% CI 0·09–0·60); the study had insufficient power to stratify that age group further.

There are several limitations to our analysis. Stratified analyses have the benefit of controlling for confounding by interactions between variables such as ethnicity, age, and time, but the disadvantage of discarding data from strata where cases from one of the two groups being compared are absent. However, we found estimates showed little sensitivity to the level of stratification used. If rates of progression from infection to symptom onset or test date differ by variant, the HRs presented might be biased. Data on hospital activity, deaths, and vaccination status are subject to reporting delays, and although we mitigated potential biases arising from these delays by using survival analysis stratified by test date, age, and residential region, residual bias might remain. NHS numbers are required to link case data to hospital attendance information and vaccination status; therefore, 89 184 people (5·4% of cases) without NHS numbers were excluded from our primary analysis. However, we have no reason to expect a strong association between the absence of NHS number and SARS-CoV-2 variant. During the study period, the omicron variant experienced a rapidly increasing incidence, whereas the delta variant was experiencing a decreasing or less rapidly increasing incidence. These trends could result in epidemic phase bias if infection severity is correlated with time from infection to test.^29^ Exploratory analysis suggested that such bias would lead to similar or lower HRs between omicron and delta cases compared with those estimated in our primary analysis. It is also unclear whether virologically confirmed cases represent a comparable fraction of underlying infections for the two variants we compared. If the proportion of omicron infections confirmed via PCR testing is lower or higher than for contemporaneous delta infections, our hospitalisation HR estimates will be over-estimates or under-estimates of the true severity of omicron relative to delta. Finally, cases of the BA.2 lineage of omicron were not considered in this analysis, because incidence of that lineage only reached substantial levels after the start of 2022, meaning insufficient data have accumulated to allow reliable severity estimates to be generated.

It is not inevitable that viral evolution leads to lower severity. The risk of hospitalisation appeared to increase when comparing delta with alpha infections^21^, ^34^, ^35^, ^36^ and when comparing alpha with previously circulating lineages.^36^, ^37^, ^38^ However, our analysis indicates that omicron is associated with a substantially lower risk of severe outcomes in adults than that of the previously dominant delta variant. Lower severity also needs to be counterbalanced against the ability of a variant to evade pre-existing immunity and thus transmit more readily within highly immune populations. We find evidence of moderate reductions in the protection vaccines provide against hospitalisation in breakthrough omicron cases compared with that of delta, and previous studies indicate substantial reductions in vaccine effectiveness against symptomatic infection.^18^ However, we find that receiving an mRNA vaccine booster dose gives over 70% protection against hospitalisation or mortality outcomes in breakthrough omicron cases; absolute vaccine effectiveness will be substantially higher once protection against infection is accounted for.^19^

We were not able to evaluate more detailed measures of relative clinical severity in hospitalised patients (such as intensive care unit admittance), but our finding that estimated severity reductions comparing omicron with delta are larger for more severe endpoints (death and hospital admission versus hospital attendance) agrees with observations that the proportion of hospitalised COVID-19 patients requiring intensive care or mechanical ventilation (or both) has been substantially lower during the omicron wave in England than the preceding delta wave.^39^ The 80% overall reduction in the intrinsic risk of death that we estimate for omicron infection compared with that of delta will make the goal of living with COVID-19 in the absence of socially and economically disruptive public health interventions substantially easier to achieve at the current time. However, it is not guaranteed that future variants will have a similarly reduced severity. It is, therefore, crucial that sufficiently detailed and systematic surveillance is maintained to allow timely detection and characterisation of new viral lineages.

## Data sharing

Although all data used in this analysis were anonymised, the individual-level nature of the data used risks individuals being identified, or being able to self-identify, if the data are released publicly. Requests for access to the underlying source data should be directed to UKHSA. The code used to analyse the data is available on GitHub.

## Declaration of interests

GD declares that his employer UK Health Security Agency (previously operating as Public Health England) received funding from GlaxoSmithKline for a research project related to influenza antiviral treatment. This preceded and had no relation to COVID-19, and GD had no role in and received no funding from the project. All other authors declare no competing interests.
