# Supplementary material for: Comparative analysis of the risks of hospitalisation and death associated with SARS-CoV-2 omicron (B.1.1.529) and delta (B.1.617.2) variants in England: a cohort study
Source: Lancet. 2022 Apr 2;399(10332):1303–12. doi: 10.1016/S0140-6736(22)00462-7 (PMC8926413; doi:10.1016/S0140-6736(22)00462-7)
Supplement: Supplementary appendix [file mmc1.pdf]

THE LANCET

Supplementary appendix

This appendix formed part of the original submission and has been peer reviewed. We post it as supplied by the authors.

Supplement to: Nyberg T, Ferguson N M, Nash S G, et al. Comparative analysis of the risks of hospitalisation and death associated with SARS-CoV-2 omicron (B.1.1.529) and delta (B.1.617.2) variants in England: a cohort study. *Lancet* 2022; published online Mar 16. https://doi.org/10.1016/S0140-6736(22)00514-1
.

Comparative analysis of the risks of hospitalisation and death associated with SARS-CoV-2 Omicron (B.1.1.529) and Delta (B.1.617.2) variants in England

Appendix

Table of Contents

p2 Figure S1. Data selection flow chart

p3 Figure S2. Positive and negative predictive values for the use of S-gene target failure (SGTF)

p4 Figure S3. Descriptive frequencies for all cases in study period

p5 Figure S4. COVID-19 cases and hospitalisations in 0-9 year age band, by SARS-CoV-2 variant

p6 Table S1. Characteristics of COVID-19 cases included and not included in the analysis

p7 Table S2. Omicron:Delta HRs for hospitalisation and mortality, overall and by age group

p8 Table S3. HR estimates for vaccination and prior infection categories

p10 Table S4: As Table S2, but splitting <10 age band into <1 year, 1-4 years, 5-9 years bands

p11 Table S5. Sensitivity analyses: estimates restricted to the subgroup of unvaccinated cases

p12 Table S6. Sensitivity analyses: effect of allowing past infection to vary by variant

p13 Table S7. Sensitivity analyses: effect of variant classification method and pillar

p14 Table S8: Sensitivity analyses: effect of stratification versus regression adjustments

p16 Table S9: Sensitivity analyses: effect of varying hospitalisation outcome definition

p17 Table S10: Sensitivity analysis: effect of epidemic phase bias

p18 Table S11: Sensitivity analysis: adjustment for under-ascertainment of past infections Omicron:Delta HRs

p19 Table S12: Sensitivity analysis: adjustment for under-ascertainment of past infections Vaccination and reinfection HRs

p21 Supplementary methods. Sensitivity analysis adjusting for under-ascertainment of past infections

p22 References

Figure S1. Data selection flow chart

Total number of COVID-19 cases during the inclusion period, n = 4,135,347

Exclusions

SARS-CoV-2 variant unknown (No whole genome sequencing, genotyping, or S gene positivity data, or only S gene positivity data after 30 December 2021), n = 2,498,193

Whole-genome-sequencing- or genotyping-confirmed SARS-CoV-2 variants other than Omicron or Delta, n = 504

Confirmed or likely Omicron or Delta cases n = 1,636,650

Exclusions

Date inconsistency: death date before specimen date, n = 90

Missing NHS number, n = 89,184

Missing data on at least one adjustment variable, n = 2,197

International travel within 14 days before positive test, n = 26,411

Received 4 or more vaccine doses, n = 660

Received 3rd vaccine dose <80 days after second dose, n = 372

Received vaccine other than AstraZeneca, Pfizer or Moderna, n = 1034

Included confirmed or likely Omicron or Delta cases, n = 1,516,702

Figure S2. Positive and negative predictive values for the use of S-gene target failure (SGTF) to classify Omicron or Delta, by date of positive test, calculated by comparison of SGTF data with results from genotyping or sequencing for cases where both data types were available. PPV was defined as the probability that cases whose specimen was associated with SGTF were confirmed to be infected with Omicron, and NPV was defined as the probability that case with S gene positive specimens were confirmed to be infected with Delta. The PPV to classify Omicron cases was estimated to be 78% at the start of the inclusion period and rose to >99% consistently after 8 December. The NPV was >99% at the start of the inclusion period but declined (due to the BA.2 S-gene positive Omicron variant) to 87% by 30 December and to 20% at the end of the inclusion period.

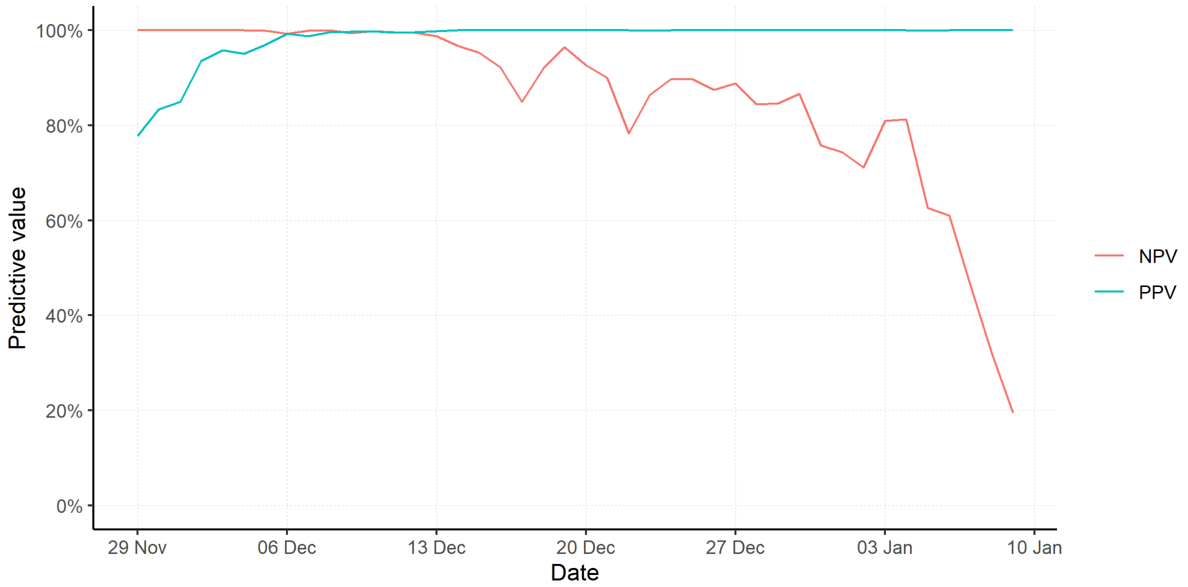

Figure S3. Descriptive frequencies for all cases identified between 29 November 2021 and 9 January 2022, including cases not fulfilling the inclusion criteria for the study: (A) Number of COVID-19 cases in total; and the corresponding (B) number of hospital admissions; and (C) number of hospital attendances (including admissions); and (D) number of hospital attendances (including admissions and diagnoses during ongoing hospital admissions), within 14 days, by variant and date of positive test.

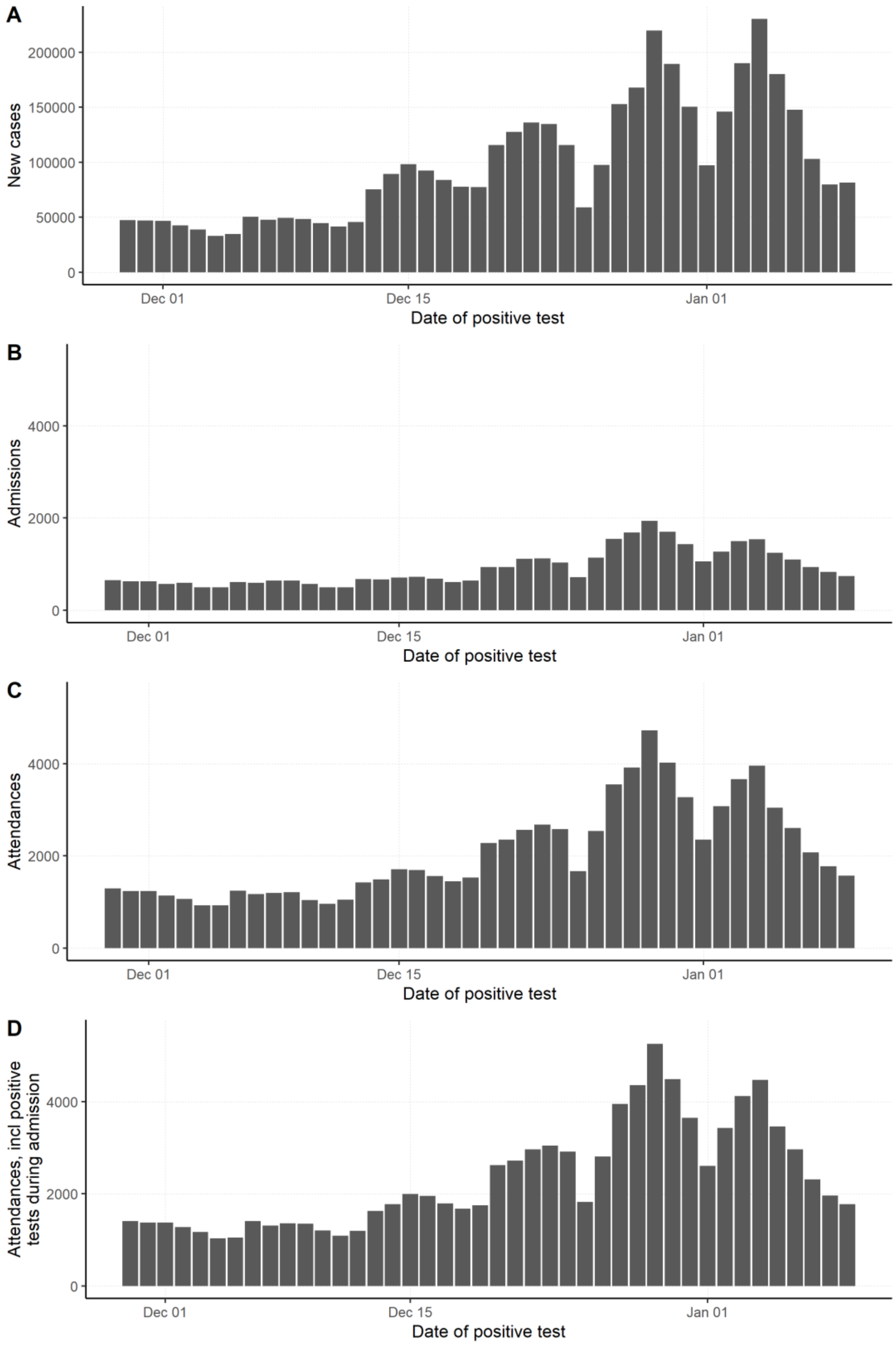

Figure S4. COVID-19 cases and hospitalisations in 0-9 year age band, by SARS-CoV-2 variant: (A) Proportion of cases by year of age within age band, (B) proportion of 0-9 year-old cases with different hospitalisation outcomes for Delta and Omicron, (C) proportion of 0-9 year-old cases with different hospitalisation outcomes for Delta and Omicron by individual year of age.

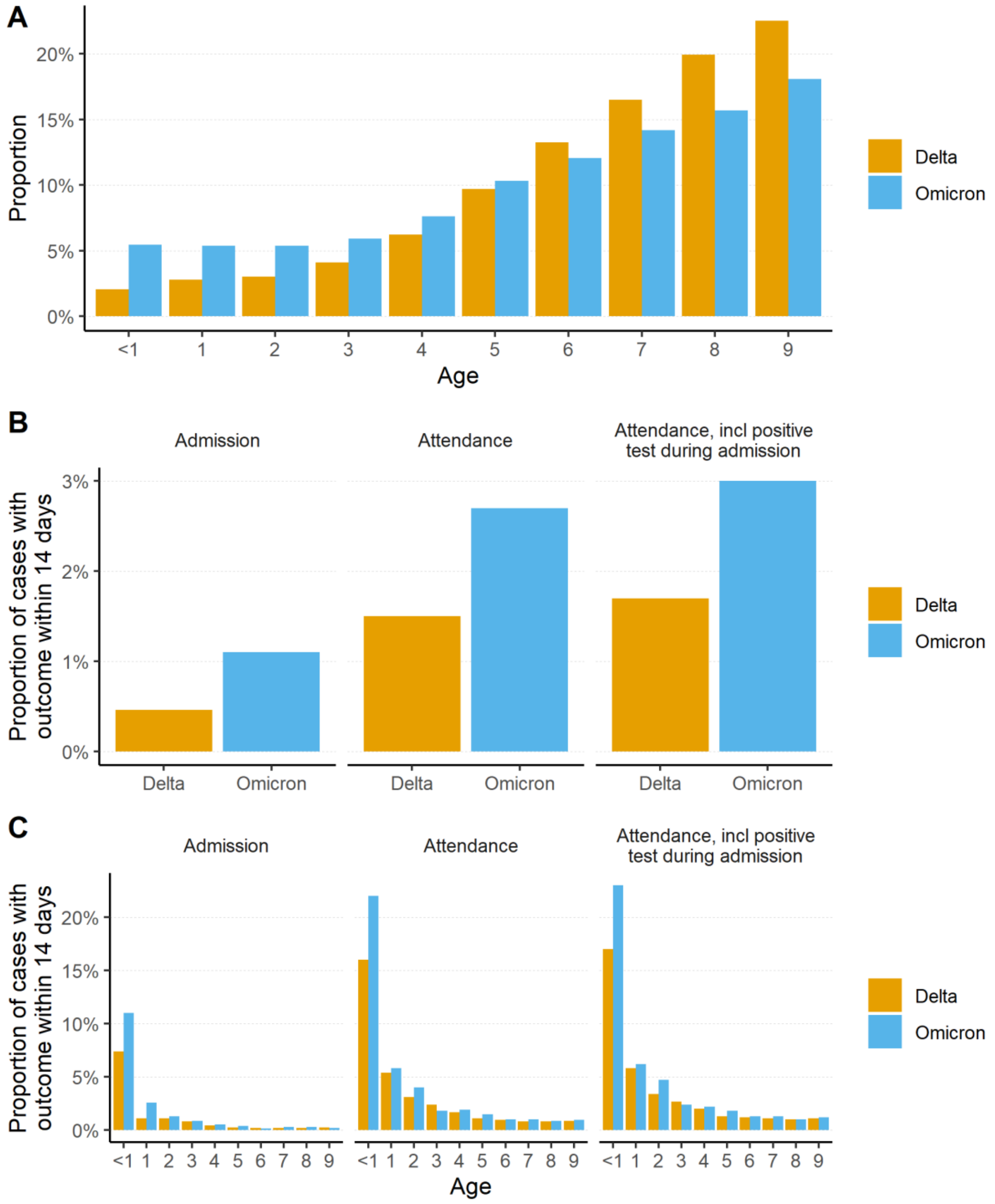

Table S1. Characteristics of COVID-19 cases included and not included in the analysis (see appendix, p. 2 for inclusion criteria).

Characteristic	Overall n (%)	Omicron n (%)	Delta n (%)	Not included n (%)
Total	1516702	1067859	448843	2618645
Age				
<10	117631 (7.8%)	43147 (4.0%)	74484 (16.6%)	198431 (7.6%)
10-19	207545 (13.7%)	119261 (11.2%)	88284 (19.7%)	353151 (13.5%)
20-29	313377 (20.7%)	265199 (24.8%)	48178 (10.7%)	562890 (21.5%)
30-39	308316 (20.3%)	228915 (21.4%)	79401 (17.7%)	515098 (19.7%)
40-49	248805 (16.4%)	167045 (15.6%)	81760 (18.2%)	396404 (15.1%)
50-59	183484 (12.1%)	134186 (12.6%)	49298 (11.0%)	315746 (12.1%)
60-69	83205 (5.5%)	64875 (6.1%)	18330 (4.1%)	157609 (6.0%)
70-79	37009 (2.4%)	31066 (2.9%)	5943 (1.3%)	75679 (2.9%)
80+	17330 (1.1%)	14165 (1.3%)	3165 (0.7%)	41588 (1.6%)
Unknown	0	0	0	2049 (0.1%)
Sex				
Female	807595 (53.2%)	573387 (53.7%)	234208 (52.2%)	1395832 (53.3%)
Male	709107 (46.8%)	494472 (46.3%)	214635 (47.8%)	1208051 (46.1%)
Unknown	0	0	0	14762 (0.6%)
Ethnicity				
White	1263101 (83.3%)	875219 (82.0%)	387882 (86.4%)	2007015 (76.6%)
Black	80921 (5.3%)	65891 (6.2%)	15030 (3.3%)	167671 (6.4%)
Indian	43720 (2.9%)	33072 (3.1%)	10648 (2.4%)	82076 (3.1%)
Pakistani or Bangladeshi	31317 (2.1%)	21864 (2.0%)	9453 (2.1%)	71296 (2.7%)
Other	77761 (5.1%)	56926 (5.3%)	20835 (4.6%)	149784 (5.7%)
Unknown	19882 (1.3%)	14887 (1.4%)	4995 (1.1%)	140803 (5.4%)
Region of residence				
East of England	141840 (9.4%)	92486 (8.7%)	49354 (11.0%)	334940 (12.8%)
London	252343 (16.6%)	204397 (19.1%)	47946 (10.7%)	548400 (20.9%)
Midlands	247866 (16.3%)	164390 (15.4%)	83476 (18.6%)	470581 (18.0%)
North East and Yorkshire	270200 (17.8%)	185596 (17.4%)	84604 (18.8%)	326718 (12.5%)
North West	255793 (16.9%)	191748 (18.0%)	64045 (14.3%)	335558 (12.8%)
South East	224973 (14.8%)	152095 (14.2%)	72878 (16.2%)	384155 (14.7%)
South West	123687 (8.2%)	77147 (7.2%)	46540 (10.4%)	188521 (7.2%)
Unknown	0	0	0	29772 (1.1%)
Index of multiple deprivation				
1st decile (most deprived)	141357 (9.3%)	97415 (9.1%)	43942 (9.8%)	244369 (9.3%)
2nd decile	148090 (9.8%)	107484 (10.1%)	40606 (9.0%)	274341 (10.5%)
3rd decile	155276 (10.2%)	113873 (10.7%)	41403 (9.2%)	283659 (10.8%)
4th decile	152424 (10.0%)	109294 (10.2%)	43130 (9.6%)	272240 (10.4%)
5th decile	150133 (9.9%)	106650 (10.0%)	43483 (9.7%)	263609 (10.1%)
6th decile	151767 (10.0%)	106112 (9.9%)	45655 (10.2%)	261359 (10.0%)
7th decile	151693 (10.0%)	105859 (9.9%)	45834 (10.2%)	249140 (9.5%)
8th decile	155589 (10.3%)	108428 (10.2%)	47161 (10.5%)	252031 (9.6%)
9th decile	155708 (10.3%)	107643 (10.1%)	48065 (10.7%)	247841 (9.5%)
10th decile (least deprived)	154665 (10.2%)	105101 (9.8%)	49564 (11.0%)	240284 (9.2%)
Unknown	0	0	0	29772 (1.1%)
Calendar week of specimen				
29 November - 5 December 2021	157414 (10.4%)	1516 (0.1%)	155898 (34.7%)	133084 (5.1%)
6 December - 12 December 2021	171374 (11.3%)	31382 (2.9%)	139992 (31.2%)	156067 (6.0%)
13 December - 19 December 2021	288549 (19.0%)	193630 (18.1%)	94919 (21.1%)	306153 (11.7%)
20 December - 26 December 2021	402599 (26.5%)	359492 (33.7%)	43107 (9.6%)	384067 (14.7%)
27 December 2021 - 2 January 2022	361392 (23.8%)	347683 (32.6%)	13709 (3.1%)	762132 (29.1%)
3 January - 9 January 2022	135374 (8.9%)	134156 (12.6%)	1218 (0.3%)	877142 (33.5%)
Vaccination status at date of specimen				
Unvaccinated	380712 (25.1%)	192426 (18.0%)	188286 (41.9%)	573805 (21.9%)
AstraZeneca, 1 dose	8757 (0.6%)	5640 (0.5%)	3117 (0.7%)	12952 (0.5%)
AstraZeneca, 2 doses	237755 (15.7%)	134262 (12.6%)	103493 (23.1%)	282063 (10.8%)
AstraZeneca, 3 doses	251224 (16.6%)	215603 (20.2%)	35621 (7.9%)	440129 (16.8%)
Pfizer/Moderna, 1 dose	89141 (5.9%)	58776 (5.5%)	30365 (6.8%)	137778 (5.3%)
Pfizer/Moderna, 2 doses	337023 (22.2%)	268430 (25.1%)	68593 (15.3%)	488328 (18.6%)
Pfizer/Moderna, 3 doses	212090 (14.0%)	192722 (18.0%)	19368 (4.3%)	399325 (15.2%)
Unknown	0	0	0	284265 (10.9%)
Reinfection status				
First infection	1407762 (92.8%)	964902 (90.4%)	442860 (98.7%)	2393834 (91.4%)
Reinfection	108940 (7.2%)	102957 (9.6%)	5983 (1.3%)	224811 (8.6%)

Table S2: Risk of hospitalisation and mortality for COVID-19 cases with Omicron compared with Delta, overall and by age group. From left to right, columns show: observed counts and proportions for three hospitalisation endpoints and death within 28 days of a positive test; unadjusted hazard ratio (HR) estimates by each age stratum; adjusted estimates derived from the primary analysis, which stratifies by vaccination category, but does not estimate vaccine effectiveness; and adjusted estimates for the unvaccinated population, from the secondary analysis, which estimated variant-specific HRs for hospitalisation or death in vaccinated groups versus unvaccinated, additionally to the Omicron vs Delta HRs.

Outcome	Age group	Omicron, n/N (%)*	Delta, n/N (%)*	HR (95% CI), Omicron vs Delta		
				Unadjusted	Adjusted for confounders†	Adjusted for confounders, estimates for unvaccinated‡
Hospital admission up to 14 days after positive test	All ages	9624/1067859 (0.90%)	7358/448843 (1.64%)	0.55 (0.53-0.56)	0.41 (0.39-0.43)	0.30 (0.28-0.32)
	<10 years	475/43147 (1.10%)	345/74484 (0.46%)	2.39 (2.08-2.74)	1.10 (0.85-1.42)	1.10 (0.85-1.42)
	10-19 years	456/119261 (0.38%)	288/88284 (0.33%)	1.17 (1.01-1.36)	0.83 (0.64-1.08)	0.78 (0.60-1.00)
	20-29 years	1593/265199 (0.60%)	642/48178 (1.33%)	0.45 (0.41-0.49)	0.55 (0.48-0.63)	0.43 (0.37-0.49)
	30-39 years	1500/228915 (0.66%)	1192/79401 (1.50%)	0.43 (0.40-0.47)	0.44 (0.39-0.50)	0.31 (0.28-0.35)
	40-49 years	987/167045 (0.59%)	1147/81760 (1.40%)	0.42 (0.39-0.46)	0.33 (0.29-0.38)	0.20 (0.17-0.23)
	50-59 years	1031/134186 (0.77%)	1175/49298 (2.38%)	0.32 (0.29-0.35)	0.26 (0.23-0.30)	0.14 (0.12-0.17)
	60-69 years	900/64875 (1.39%)	970/18330 (5.29%)	0.26 (0.23-0.28)	0.25 (0.21-0.30)	0.14 (0.12-0.16)
	70-79 years	1108/31066 (3.57%)	795/5943 (13.4%)	0.25 (0.23-0.28)	0.36 (0.30-0.43)	0.20 (0.17-0.24)
	≥80 years	1574/14165 (11.1%)	804/3165 (25.4%)	0.40 (0.37-0.44)	0.47 (0.40-0.56)	0.33 (0.28-0.39)
Any hospital attendance (including admission) up to 14 days after positive test	All ages	22798/1067859 (2.13%)	13519/448843 (3.01%)	0.71 (0.69-0.72)	0.56 (0.54-0.58)	0.41 (0.39-0.43)
	<10 years	1158/43147 (2.68%)	1111/74484 (1.49%)	1.81 (1.67-1.97)	1.03 (0.89-1.19)	1.03 (0.89-1.19)
	10-19 years	1390/119261 (1.17%)	933/88284 (1.06%)	1.10 (1.02-1.20)	0.89 (0.76-1.03)	0.84 (0.73-0.98)
	20-29 years	4758/265199 (1.79%)	1448/48178 (3.01%)	0.59 (0.56-0.63)	0.67 (0.62-0.74)	0.52 (0.48-0.57)
	30-39 years	4406/228915 (1.92%)	2596/79401 (3.27%)	0.58 (0.56-0.61)	0.57 (0.52-0.61)	0.40 (0.37-0.43)
	40-49 years	2949/167045 (1.77%)	2230/81760 (2.73%)	0.64 (0.61-0.68)	0.54 (0.49-0.59)	0.32 (0.29-0.35)
	50-59 years	2632/134186 (1.96%)	1938/49298 (3.93%)	0.49 (0.47-0.52)	0.42 (0.38-0.46)	0.22 (0.20-0.25)
	60-69 years	1801/64875 (2.78%)	1377/18330 (7.51%)	0.36 (0.34-0.39)	0.32 (0.28-0.37)	0.16 (0.14-0.18)
	70-79 years	1681/31066 (5.41%)	963/5943 (16.2%)	0.31 (0.29-0.34)	0.42 (0.36-0.50)	0.19 (0.16-0.22)
	≥80 years	2023/14165 (14.3%)	923/3165 (29.2%)	0.45 (0.41-0.48)	0.49 (0.42-0.58)	0.27 (0.23-0.31)
Any hospital attendance (including admission) up to 14 days after positive test, or positive test during hospital stay	All ages	25815/1067859 (2.42%)	14739/448843 (3.28%)	0.73 (0.72-0.75)	0.59 (0.57-0.61)	0.44 (0.42-0.46)
	<10 years	1304/43147 (3.02%)	1300/74484 (1.75%)	1.75 (1.62-1.89)	1.04 (0.91-1.19)	1.04 (0.91-1.19)
	10-19 years	1749/119261 (1.47%)	1203/88284 (1.36%)	1.08 (1.00-1.16)	0.88 (0.77-1.00)	0.85 (0.75-0.97)
	20-29 years	5593/265199 (2.11%)	1577/48178 (3.27%)	0.64 (0.61-0.68)	0.70 (0.65-0.77)	0.55 (0.51-0.60)
	30-39 years	5000/228915 (2.18%)	2788/79401 (3.51%)	0.62 (0.59-0.65)	0.59 (0.55-0.63)	0.41 (0.38-0.45)
	40-49 years	3315/167045 (1.98%)	2375/81760 (2.90%)	0.68 (0.65-0.72)	0.57 (0.52-0.62)	0.34 (0.31-0.37)
	50-59 years	2920/134186 (2.18%)	2044/49298 (4.15%)	0.52 (0.49-0.55)	0.44 (0.40-0.49)	0.24 (0.22-0.26)
	60-69 years	1976/64875 (3.05%)	1447/18330 (7.89%)	0.38 (0.35-0.40)	0.34 (0.30-0.39)	0.17 (0.15-0.19)
	70-79 years	1802/31066 (5.80%)	1007/5943 (16.9%)	0.32 (0.30-0.35)	0.45 (0.38-0.52)	0.20 (0.18-0.24)
	≥80 years	2156/14165 (15.2%)	998/3165 (31.5%)	0.43 (0.40-0.47)	0.53 (0.45-0.62)	0.29 (0.25-0.34)
Death within 28 days after positive test	All ages	1225/1067859 (0.11%)	1205/448843 (0.27%)	0.44 (0.41-0.48)	0.31 (0.26-0.37)	0.20 (0.16-0.25)
	<10 years	2/43147 (0.005%)	1/74484 (0.001%)	--§	--§	--§
	10-19 years	1/119261 (0.001%)	1/88284 (0.001%)	--§	--§	--§
	20-29 years	5/265199 (0.002%)	2/48178 (0.004%)	--§	--§	--§
	30-39 years	11/228915 (0.005%)	28/79401 (0.04%)	0.14 (0.07-0.28)	0.28 (0.07-1.04)	0.13 (0.04-0.46)
	40-49 years	25/167045 (0.01%)	43/81760 (0.05%)	0.29 (0.18-0.48)	0.25 (0.11-0.55)	0.11 (0.05-0.24)
	50-59 years	71/134186 (0.05%)	128/49298 (0.26%)	0.21 (0.16-0.28)	0.16 (0.09-0.27)	0.07 (0.04-0.12)
	60-69 years	128/64875 (0.20%)	206/18330 (1.12%)	0.19 (0.15-0.23)	0.22 (0.15-0.34)	0.11 (0.07-0.16)
	70-79 years	257/31066 (0.83%)	294/5943 (4.95%)	0.17 (0.14-0.20)	0.26 (0.18-0.36)	0.16 (0.11-0.22)
	≥80 years	725/14165 (5.12%)	502/3165 (15.9%)	0.32 (0.29-0.36)	0.46 (0.36-0.58)	0.36 (0.27-0.48)

* These crude descriptive frequencies are unadjusted for age and other confounders, and so are not directly comparable between groups.

† Based on models stratified for calendar date, region, age group, ethnicity and vaccination status, and using regression adjustment for within-age-group linear age, sex, index of multiple deprivation and vaccine-status-specific past infection status.

‡ Adjusted HR estimates for unvaccinated cases. Based on models stratified for calendar date, region, age group and ethnicity, and using regression adjustment for within-age-group linear age, sex, index of multiple deprivation and explicit modelling of the effects of past infection and vaccination history. Variant-specific HRs for vaccinated compared with unvaccinated cases are shown in Figure 2 and appendix p. 6-7.

§ Not estimated due to small numbers.

Table S3. HR estimates for vaccination and prior infection categories for the secondary analysis model used to estimate age-specific hazard ratios (HRs) for Omicron versus Delta and variant-specific vaccine effectiveness. Corresponding age-specific hazard ratios for Omicron vs Delta cases in unvaccinated individuals are shown in the rightmost column of Table 1. HRs for vaccination categories are versus unvaccinated cases, those for past infection categories are versus cases with no documented past infection. Vaccination categories are stratified by vaccine given for doses 1 and 2, number of doses received (dose 3 was always Pfizer or Moderna booster), and weeks elapsed from last dose to positive SARS-CoV-2 specimen date.

Variable	HR (95% CI)			
	Hospital admission up to 14 days after positive test	Any hospital attendance (including admission) up to 14 days after positive test	Any hospital attendance (including admission) up to 14 days after positive test, or positive test during hospital stay	Death within 28 days after positive test
Delta: AstraZeneca, dose 1, any time*	0.55 (0.46-0.66)	0.65 (0.57-0.75)	0.69 (0.60-0.78)	0.57 (0.37-0.88)
Delta: AstraZeneca, dose 2, <2 weeks	0.17 (0.08-0.38)	0.24 (0.13-0.45)	0.23 (0.13-0.44)	--
Delta: AstraZeneca, dose 2, 2-7 weeks	0.18 (0.07-0.50)	0.22 (0.10-0.49)	0.21 (0.09-0.48)	0.31 (0.04-2.47)
Delta: AstraZeneca, dose 2, 8-11 weeks	0.61 (0.28-1.31)	0.51 (0.26-0.99)	0.51 (0.26-0.99)	0.37 (0.05-2.87)
Delta: AstraZeneca, dose 2, 12-15 weeks	0.32 (0.17-0.59)	0.32 (0.19-0.51)	0.33 (0.21-0.53)	--
Delta: AstraZeneca, dose 2, 16-19 weeks	0.14 (0.10-0.21)	0.24 (0.19-0.30)	0.27 (0.21-0.33)	0.23 (0.07-0.78)
Delta: AstraZeneca, dose 2, 20+ weeks	0.22 (0.20-0.23)	0.28 (0.27-0.29)	0.30 (0.29-0.32)	0.27 (0.23-0.33)
Delta: AstraZeneca, dose 3, <2 weeks	0.11 (0.09-0.12)	0.15 (0.14-0.17)	0.16 (0.15-0.18)	0.11 (0.08-0.16)
Delta: AstraZeneca, dose 3, 2-7 weeks	0.13 (0.12-0.16)	0.18 (0.16-0.20)	0.19 (0.17-0.21)	0.13 (0.10-0.17)
Delta: AstraZeneca, dose 3, 8-11 weeks	0.15 (0.12-0.19)	0.22 (0.18-0.26)	0.24 (0.20-0.28)	0.16 (0.11-0.23)
Delta: AstraZeneca, dose 3, 12+ weeks	0.30 (0.17-0.55)	0.35 (0.21-0.60)	0.37 (0.22-0.62)	0.06 (0.01-0.48)
Delta: Pfizer/Moderna, dose 1, <2 weeks	0.56 (0.41-0.78)	0.68 (0.55-0.84)	0.70 (0.58-0.86)	0.42 (0.13-1.43)
Delta: Pfizer/Moderna, dose 1, 2-7 weeks	0.35 (0.25-0.47)	0.46 (0.38-0.56)	0.50 (0.42-0.59)	0.79 (0.29-2.14)
Delta: Pfizer/Moderna, dose 1, 8-11 weeks	0.39 (0.26-0.59)	0.48 (0.37-0.61)	0.58 (0.47-0.71)	0.21 (0.03-1.57)
Delta: Pfizer/Moderna, dose 1, 12+ weeks	0.37 (0.30-0.45)	0.44 (0.39-0.50)	0.47 (0.42-0.53)	0.39 (0.20-0.76)
Delta: Pfizer/Moderna, dose 2, <2 weeks	0.19 (0.10-0.37)	0.34 (0.23-0.49)	0.33 (0.23-0.48)	--
Delta: Pfizer/Moderna, dose 2, 2-7 weeks	0.45 (0.29-0.70)	0.43 (0.31-0.59)	0.48 (0.35-0.65)	0.11 (0.01-0.96)
Delta: Pfizer/Moderna, dose 2, 8-11 weeks	0.24 (0.16-0.37)	0.32 (0.25-0.42)	0.36 (0.28-0.46)	0.19 (0.05-0.80)
Delta: Pfizer/Moderna, dose 2, 12-15 weeks	0.14 (0.11-0.19)	0.22 (0.18-0.26)	0.25 (0.21-0.29)	--
Delta: Pfizer/Moderna, dose 2, 16-19 weeks	0.11 (0.09-0.13)	0.18 (0.16-0.21)	0.20 (0.18-0.23)	0.08 (0.02-0.34)
Delta: Pfizer/Moderna, dose 2, 20+ weeks	0.19 (0.17-0.22)	0.25 (0.23-0.28)	0.27 (0.25-0.29)	0.30 (0.24-0.38)
Delta: Pfizer/Moderna, dose 3, <2 weeks	0.14 (0.11-0.18)	0.17 (0.15-0.21)	0.18 (0.16-0.22)	0.16 (0.10-0.25)
Delta: Pfizer/Moderna, dose 3, 2-7 weeks	0.13 (0.11-0.15)	0.19 (0.17-0.21)	0.20 (0.18-0.22)	0.14 (0.11-0.19)
Delta: Pfizer/Moderna, dose 3, 8-11 weeks	0.12 (0.10-0.14)	0.17 (0.15-0.19)	0.17 (0.15-0.20)	0.12 (0.09-0.15)
Delta: Pfizer/Moderna, dose 3, 12+ weeks	0.17 (0.11-0.25)	0.20 (0.14-0.28)	0.20 (0.14-0.29)	0.13 (0.07-0.26)
Omicron: AstraZeneca, dose 1, any time*	1.18 (1.01-1.39)	1.27 (1.14-1.43)	1.27 (1.14-1.42)	1.06 (0.69-1.64)
Omicron: AstraZeneca, dose 2, <2 weeks	0.61 (0.33-1.14)	0.66 (0.43-1.02)	0.61 (0.40-0.94)	--
Omicron: AstraZeneca, dose 2, 2-7 weeks	0.40 (0.24-0.67)	0.53 (0.38-0.73)	0.50 (0.36-0.70)	0.16 (0.02-1.15)
Omicron: AstraZeneca, dose 2, 8-11 weeks	0.65 (0.41-1.04)	0.68 (0.47-0.99)	0.72 (0.51-1.02)	0.79 (0.34-1.83)
Omicron: AstraZeneca, dose 2, 12-15 weeks	0.50 (0.28-0.89)	0.59 (0.40-0.88)	0.62 (0.42-0.90)	0.59 (0.18-1.98)
Omicron: AstraZeneca, dose 2, 16-19 weeks	0.54 (0.37-0.79)	0.65 (0.52-0.83)	0.69 (0.55-0.86)	1.08 (0.38-3.14)
Omicron: AstraZeneca, dose 2, 20+ weeks	0.55 (0.51-0.59)	0.62 (0.59-0.65)	0.65 (0.62-0.68)	0.83 (0.66-1.03)
Omicron: AstraZeneca, dose 3, <2 weeks	0.29 (0.26-0.33)	0.38 (0.35-0.40)	0.40 (0.37-0.43)	0.26 (0.17-0.40)
Omicron: AstraZeneca, dose 3, 2-7 weeks	0.22 (0.20-0.24)	0.35 (0.33-0.37)	0.37 (0.35-0.39)	0.18 (0.14-0.23)
Omicron: AstraZeneca, dose 3, 8-11 weeks	0.21 (0.19-0.24)	0.43 (0.40-0.46)	0.44 (0.41-0.47)	0.16 (0.12-0.20)
Omicron: AstraZeneca, dose 3, 12+ weeks	0.23 (0.19-0.28)	0.48 (0.42-0.54)	0.48 (0.42-0.55)	0.23 (0.16-0.33)
Omicron: Pfizer/Moderna, dose 1, <2 weeks	0.70 (0.53-0.92)	0.75 (0.63-0.90)	0.74 (0.62-0.88)	0.60 (0.21-1.70)
Omicron: Pfizer/Moderna, dose 1, 2-7 weeks	0.48 (0.38-0.60)	0.61 (0.53-0.70)	0.60 (0.53-0.68)	0.13 (0.02-0.93)
Omicron: Pfizer/Moderna, dose 1, 8-11 weeks	0.51 (0.38-0.68)	0.58 (0.49-0.69)	0.60 (0.51-0.69)	0.68 (0.27-1.72)
Omicron: Pfizer/Moderna, dose 1, 12+ weeks	0.65 (0.56-0.75)	0.72 (0.66-0.79)	0.75 (0.68-0.81)	0.58 (0.28-1.23)
Omicron: Pfizer/Moderna, dose 2, <2 weeks	0.54 (0.39-0.75)	0.62 (0.51-0.75)	0.60 (0.50-0.72)	1.37 (0.30-6.30)
Omicron: Pfizer/Moderna, dose 2, 2-7 weeks	0.40 (0.31-0.51)	0.49 (0.42-0.57)	0.52 (0.46-0.60)	0.97 (0.44-2.14)
Omicron: Pfizer/Moderna, dose 2, 8-11 weeks	0.38 (0.30-0.48)	0.53 (0.46-0.60)	0.54 (0.48-0.61)	0.05 (0.01-0.43)
Omicron: Pfizer/Moderna, dose 2, 12-15 weeks	0.31 (0.27-0.37)	0.39 (0.35-0.43)	0.42 (0.38-0.45)	0.19 (0.05-0.77)
Omicron: Pfizer/Moderna, dose 2, 16-19 weeks	0.25 (0.22-0.28)	0.35 (0.32-0.37)	0.38 (0.35-0.40)	0.09 (0.01-0.69)
Omicron: Pfizer/Moderna, dose 2, 20+ weeks	0.43 (0.39-0.46)	0.53 (0.50-0.56)	0.54 (0.51-0.57)	0.81 (0.62-1.04)

Omicron: Pfizer/Moderna, dose 3, <2 weeks	0.23 (0.20-0.26)	0.32 (0.30-0.35)	0.36 (0.33-0.39)	0.28 (0.16-0.51)
Omicron: Pfizer/Moderna, dose 3, 2-7 weeks	0.26 (0.23-0.29)	0.43 (0.40-0.47)	0.45 (0.42-0.48)	0.22 (0.16-0.30)
Omicron: Pfizer/Moderna, dose 3, 8-11 weeks	0.22 (0.20-0.24)	0.40 (0.38-0.43)	0.42 (0.39-0.45)	0.15 (0.12-0.20)
Omicron: Pfizer/Moderna, dose 3, 12+ weeks	0.19 (0.17-0.22)	0.38 (0.35-0.42)	0.39 (0.36-0.43)	0.13 (0.10-0.17)
Past infection vs no past infection, unvaccinated	0.55 (0.48-0.63)	0.68 (0.62-0.74)	0.72 (0.67-0.78)	0.18 (0.06-0.57)
Past infection vs no past infection, vaccinated	0.96 (0.88-1.04)	1.03 (0.98-1.09)	1.04 (0.99-1.09)	0.47 (0.32-0.68)

* Due to small numbers, all cases who had received a single dose of the AstraZeneca vaccine are grouped together.

Table S4: As Table S2, but showing HRs for Omicron versus Delta cases, by the age bands <1 year, 1-4 years, 5-9 years, and 10-year bands subsequently.

Outcome	Age group	Omicron, n/N (%)*	Delta, n/N (%)*	HR (95% CI), Omicron vs Delta		
				Unadjusted	Adjusted for confounders†	Adjusted for confounders, estimates for unvaccinated‡
Hospital admission up to 14 days after positive test	<1 year	260/2346 (11.1%)	112/1517 (7.38%)	1.54 (1.23-1.92)	1.36 (0.87-2.12)	1.35 (0.87-2.11)
	1-4 years	131/10475 (1.25%)	93/11981 (0.78%)	1.62 (1.24-2.11)	0.76 (0.47-1.23)	0.76 (0.47-1.23)
	5-9 years	84/30326 (0.28%)	140/60986 (0.23%)	1.21 (0.92-1.58)	1.04 (0.63-1.70)	1.04 (0.63-1.70)
	10-19 years	456/119261 (0.38%)	288/88284 (0.33%)	1.17 (1.01-1.36)	0.83 (0.64-1.08)	0.78 (0.60-1.00)
	20-29 years	1593/265199 (0.60%)	642/48178 (1.33%)	0.45 (0.41-0.49)	0.55 (0.48-0.63)	0.43 (0.37-0.49)
	30-39 years	1500/228915 (0.66%)	1192/79401 (1.50%)	0.43 (0.40-0.47)	0.44 (0.39-0.50)	0.31 (0.28-0.35)
	40-49 years	987/167045 (0.59%)	1147/81760 (1.40%)	0.42 (0.39-0.46)	0.33 (0.29-0.38)	0.20 (0.17-0.23)
	50-59 years	1031/134186 (0.77%)	1175/49298 (2.38%)	0.32 (0.29-0.35)	0.26 (0.23-0.30)	0.14 (0.12-0.17)
	60-69 years	900/64875 (1.39%)	970/18330 (5.29%)	0.26 (0.23-0.28)	0.25 (0.21-0.30)	0.14 (0.12-0.16)
	70-79 years	1108/31066 (3.57%)	795/5943 (13.4%)	0.25 (0.23-0.28)	0.36 (0.30-0.43)	0.20 (0.17-0.24)
	≥80 years	1574/14165 (11.1%)	804/3165 (25.4%)	0.40 (0.37-0.44)	0.47 (0.40-0.56)	0.33 (0.28-0.39)
Any hospital attendance (including admission) up to 14 days after positive test	<1 year	511/2346 (21.78%)	242/1517 (15.95%)	1.43 (1.23-1.67)	1.30 (0.96-1.76)	1.29 (0.96-1.75)
	1-4 years	333/10475 (3.18%)	335/11981 (2.80%)	1.14 (0.98-1.33)	0.93 (0.71-1.20)	0.93 (0.71-1.20)
	5-9 years	314/30326 (1.04%)	534/60986 (0.88%)	1.18 (1.03-1.36)	0.99 (0.78-1.25)	0.99 (0.78-1.25)
	10-19 years	1390/119261 (1.17%)	933/88284 (1.06%)	1.10 (1.02-1.20)	0.89 (0.76-1.03)	0.84 (0.73-0.98)
	20-29 years	4758/265199 (1.79%)	1448/48178 (3.01%)	0.59 (0.56-0.63)	0.67 (0.62-0.74)	0.52 (0.48-0.57)
	30-39 years	4406/228915 (1.92%)	2596/79401 (3.27%)	0.58 (0.56-0.61)	0.57 (0.52-0.61)	0.40 (0.37-0.43)
	40-49 years	2949/167045 (1.77%)	2230/81760 (2.73%)	0.64 (0.61-0.68)	0.54 (0.49-0.59)	0.32 (0.29-0.35)
	50-59 years	2632/134186 (1.96%)	1938/49298 (3.93%)	0.49 (0.47-0.52)	0.42 (0.38-0.46)	0.22 (0.20-0.25)
	60-69 years	1801/64875 (2.78%)	1377/18330 (7.51%)	0.36 (0.34-0.39)	0.32 (0.28-0.37)	0.16 (0.14-0.18)
	70-79 years	1681/31066 (5.41%)	963/5943 (16.2%)	0.31 (0.29-0.34)	0.42 (0.36-0.50)	0.19 (0.16-0.22)
	≥80 years	2023/14165 (14.3%)	923/3165 (29.2%)	0.45 (0.41-0.48)	0.49 (0.42-0.58)	0.27 (0.23-0.31)
Any hospital attendance (including admission) up to 14 days after positive test, or positive test during hospital stay	<1 year	528/2346 (22.51%)	251/1517 (16.55%)	1.43 (1.23-1.66)	1.34 (1.00-1.81)	1.33 (0.99-1.80)
	1-4 years	387/10475 (3.69%)	373/11981 (3.11%)	1.19 (1.04-1.38)	1.00 (0.78-1.28)	1.00 (0.78-1.28)
	5-9 years	389/30326 (1.28%)	676/60986 (1.11%)	1.16 (1.02-1.31)	0.96 (0.78-1.19)	0.96 (0.78-1.19)
	10-19 years	1749/119261 (1.47%)	1203/88284 (1.36%)	1.08 (1.00-1.16)	0.88 (0.77-1.00)	0.85 (0.75-0.97)
	20-29 years	5593/265199 (2.11%)	1577/48178 (3.27%)	0.64 (0.61-0.68)	0.70 (0.65-0.77)	0.55 (0.51-0.60)
	30-39 years	5000/228915 (2.18%)	2788/79401 (3.51%)	0.62 (0.59-0.65)	0.59 (0.55-0.63)	0.41 (0.38-0.45)
	40-49 years	3315/167045 (1.98%)	2375/81760 (2.90%)	0.68 (0.65-0.72)	0.57 (0.52-0.62)	0.34 (0.31-0.37)
	50-59 years	2920/134186 (2.18%)	2044/49298 (4.15%)	0.52 (0.49-0.55)	0.44 (0.40-0.49)	0.24 (0.22-0.26)
	60-69 years	1976/64875 (3.05%)	1447/18330 (7.89%)	0.38 (0.35-0.40)	0.34 (0.30-0.39)	0.17 (0.15-0.19)
	70-79 years	1802/31066 (5.80%)	1007/5943 (16.9%)	0.32 (0.30-0.35)	0.45 (0.38-0.52)	0.20 (0.18-0.24)
	≥80 years	2156/14165 (15.2%)	998/3165 (31.5%)	0.43 (0.40-0.47)	0.53 (0.45-0.62)	0.29 (0.25-0.34)
Death within 28 days after positive test	<1 year	2/2346 (0.09%)	0/1517 (0.00%)	--§	--§	--§
	1-4 years	0/10475 (0%)	1/11981 (0.01%)	--§	--§	--§
	5-9 years	0/30326 (0%)	0/60986 (0%)	--§	--§	--§
	10-19 years	1/119261 (0.001%)	1/88284 (0.001%)	--§	--§	--§
	20-29 years	5/265199 (0.002%)	2/48178 (0.004%)	--§	--§	--§
	30-39 years	11/228915 (0.005%)	28/79401 (0.04%)	0.14 (0.07-0.28)	0.28 (0.07-1.04)	0.13 (0.04-0.46)
	40-49 years	25/167045 (0.01%)	43/81760 (0.05%)	0.29 (0.18-0.48)	0.25 (0.11-0.55)	0.11 (0.05-0.24)
	50-59 years	71/134186 (0.05%)	128/49298 (0.26%)	0.21 (0.16-0.28)	0.16 (0.09-0.27)	0.07 (0.04-0.12)
	60-69 years	128/64875 (0.20%)	206/18330 (1.12%)	0.19 (0.15-0.23)	0.22 (0.15-0.34)	0.11 (0.07-0.16)
	70-79 years	257/31066 (0.83%)	294/5943 (4.95%)	0.17 (0.15-0.20)	0.26 (0.18-0.36)	0.16 (0.11-0.22)
	≥80 years	725/14165 (5.12%)	502/3165 (15.9%)	0.32 (0.29-0.36)	0.46 (0.36-0.58)	0.36 (0.27-0.48)

* These crude descriptive frequencies are unadjusted for age and other confounders, and so are not directly comparable between groups.
† Based on models stratified for calendar date, region, age group, ethnicity and vaccination status, and using regression adjustment for within-age-group linear age, sex, index of multiple deprivation and vaccine-status-specific past infection status.
‡ Adjusted HR estimates for unvaccinated cases. Based on models stratified for calendar date, region, age group and ethnicity, and using regression adjustment for within-age-group linear age, sex, index of multiple deprivation and explicit modelling of the effects of past infection and vaccination history.
§ Not estimated due to small numbers.

Table S5. Sensitivity analyses: adjusted HR estimates for Omicron vs Delta, restricted to the subgroup of unvaccinated cases.

Outcome	Age group	HR (95% CI), unvaccinated Omicron vs Delta Adjusted for confounders*
Hospital admission within 14 days after positive test	Overall	0.33 (0.31-0.36)
	<10 years	1.09 (0.84-1.41)
	10-19 years	0.68 (0.50-0.92)
	20-29 years	0.51 (0.42-0.62)
	30-39 years	0.31 (0.26-0.37)
	40-49 years	0.18 (0.14-0.22)
	50-59 years	0.13 (0.10-0.16)
	60-69 years	0.18 (0.14-0.25)
	70-79 years	0.28 (0.19-0.42)
	≥80 years	0.30 (0.20-0.46)
Any hospital attendance (including admission) within 14 days after positive test	Overall	0.47 (0.44-0.49)
	<10 years	1.02 (0.89-1.18)
	10-19 years	0.85 (0.71-1.03)
	20-29 years	0.62 (0.54-0.70)
	30-39 years	0.38 (0.34-0.43)
	40-49 years	0.28 (0.24-0.33)
	50-59 years	0.19 (0.16-0.24)
	60-69 years	0.20 (0.16-0.26)
	70-79 years	0.34 (0.23-0.48)
	≥80 years	0.35 (0.24-0.52)
Any hospital attendance (including admission) within 14 days after positive test, or positive test during hospital admission	Overall	0.50 (0.47-0.53)
	<10 years	1.04 (0.90-1.18)
	10-19 years	0.87 (0.73-1.02)
	20-29 years	0.64 (0.57-0.73)
	30-39 years	0.40 (0.36-0.45)
	40-49 years	0.31 (0.26-0.36)
	50-59 years	0.21 (0.17-0.25)
	60-69 years	0.22 (0.17-0.28)
	70-79 years	0.34 (0.24-0.49)
	≥80 years	0.45 (0.30-0.65)
Death within 28 days after positive test	Overall	0.21 (0.15-0.29)
	<10 years	--†
	10-19 years	--†
	20-29 years	--†
	30-39 years	0.09 (0.01-0.64)
	40-49 years	0.24 (0.08-0.74)
	50-59 years	0.19 (0.09-0.40)
	60-69 years	0.22 (0.11-0.46)
	70-79 years	0.18 (0.09-0.37)
	≥80 years	0.27 (0.14-0.52)

* Restricted to unvaccinated subgroup. Based on models stratified for calendar date, region, age group and ethnicity; and using regression adjustment for within-age-group linear age, sex, index of multiple deprivation and reinfection status.
† Not estimated due to small numbers.

Table S6. Sensitivity analyses: effect of allowing past infection to vary by variant. HR estimates for cases with at least one known past infection compared with cases with no known past infection shown, by SARS-CoV-2 variant.

Outcome	HR (95% CI)*, past infection versus no past infection	
	Omicron	Delta
Hospital admission up to 14 days after positive test	0.82 (0.76-0.89)	0.69 (0.55-0.88)
Any hospital attendance (including admission) up to 14 days after positive test	0.93 (0.88-0.97)	0.84 (0.72-0.99)
Any hospital attendance (including admission) up to 14 days after positive test, or positive test during hospital stay	0.94 (0.90-0.99)	0.86 (0.74-1.01)
Death within 28 days after positive test	0.34 (0.22-0.54)	0.48 (0.21-1.09)

* Based on model stratified for calendar date, region, age group, ethnicity and vaccination status; and using regression adjustment for within-age-group linear age, sex, index of multiple deprivation and reinfection status.

Table S7. Sensitivity analyses: effect of variant classification method and pillar. Age-specific adjusted hazard ratio (HR) estimates for hospitalisation and mortality outcomes for cases with the Omicron compared to the Delta variant, in three subgroups of cases: variant identified via whole genome sequencing; variant identified via sequencing or provisional genotyping; cases identified via Pillar 2 community testing alone. Pillar 2 only results were comparable to the primary analysis, while HR estimates were higher for the sequence-only analysis than the primary analysis, and lower for the genotyping-only analysis than the primary analysis.

Outcome	Age group	HR (95% CI)*			
		Primary analysis	Subgroup		
			Variant classification by whole-genome sequencing	Variant classification by whole-genome sequencing or genotyping	Identified through the Pillar 2 community testing programme
Hospital admission up to 14 days after positive test	<10 years	1.10 (0.85-1.42)	1.33 (0.82-2.18)	0.81 (0.59-1.11)	0.97 (0.64-1.46)
	10-19 years	0.83 (0.64-1.08)	0.76 (0.46-1.25)	0.56 (0.39-0.81)	0.89 (0.63-1.25)
	20-29 years	0.55 (0.48-0.63)	0.70 (0.53-0.92)	0.40 (0.33-0.49)	0.48 (0.40-0.57)
	30-39 years	0.44 (0.39-0.50)	0.53 (0.41-0.67)	0.28 (0.24-0.34)	0.47 (0.40-0.55)
	40-49 years	0.33 (0.29-0.38)	0.42 (0.32-0.57)	0.20 (0.17-0.25)	0.38 (0.32-0.46)
	50-59 years	0.26 (0.23-0.30)	0.43 (0.32-0.57)	0.17 (0.14-0.21)	0.32 (0.26-0.40)
	60-69 years	0.25 (0.21-0.30)	0.33 (0.23-0.47)	0.22 (0.17-0.27)	0.27 (0.20-0.36)
	70-79 years	0.36 (0.30-0.43)	0.40 (0.27-0.59)	0.33 (0.26-0.41)	0.38 (0.27-0.53)
	≥80 years	0.47 (0.40-0.56)	0.57 (0.42-0.79)	0.45 (0.37-0.55)	0.43 (0.30-0.61)
Any hospital attendance (including admission) up to 14 days after positive test	<10 years	1.03 (0.89-1.19)	1.16 (0.85-1.59)	0.77 (0.62-0.94)	1.11 (0.93-1.33)
	10-19 years	0.89 (0.76-1.03)	0.87 (0.63-1.22)	0.66 (0.52-0.84)	0.93 (0.78-1.11)
	20-29 years	0.67 (0.62-0.74)	0.83 (0.69-1.00)	0.56 (0.49-0.64)	0.66 (0.59-0.74)
	30-39 years	0.57 (0.52-0.61)	0.62 (0.53-0.74)	0.39 (0.34-0.43)	0.60 (0.55-0.66)
	40-49 years	0.54 (0.49-0.59)	0.58 (0.47-0.70)	0.34 (0.30-0.39)	0.61 (0.55-0.69)
	50-59 years	0.42 (0.38-0.46)	0.51 (0.42-0.64)	0.25 (0.22-0.29)	0.53 (0.46-0.61)
	60-69 years	0.32 (0.28-0.37)	0.36 (0.27-0.48)	0.22 (0.18-0.27)	0.39 (0.32-0.48)
	70-79 years	0.42 (0.36-0.50)	0.48 (0.34-0.66)	0.36 (0.30-0.44)	0.55 (0.42-0.72)
	≥80 years	0.49 (0.42-0.58)	0.58 (0.43-0.77)	0.46 (0.39-0.56)	0.44 (0.32-0.61)
Any hospital attendance (including admission) up to 14 days after positive test, or positive test during hospital stay	<10 years	1.04 (0.91-1.19)	1.19 (0.89-1.61)	0.79 (0.65-0.97)	1.10 (0.93-1.29)
	10-19 years	0.88 (0.77-1.00)	0.91 (0.68-1.21)	0.72 (0.58-0.88)	0.90 (0.77-1.04)
	20-29 years	0.70 (0.65-0.77)	0.81 (0.68-0.96)	0.56 (0.49-0.64)	0.71 (0.64-0.78)
	30-39 years	0.59 (0.55-0.63)	0.64 (0.55-0.75)	0.41 (0.37-0.46)	0.62 (0.57-0.68)
	40-49 years	0.57 (0.52-0.62)	0.64 (0.53-0.77)	0.39 (0.34-0.45)	0.64 (0.58-0.72)
	50-59 years	0.44 (0.40-0.49)	0.54 (0.44-0.67)	0.28 (0.24-0.32)	0.56 (0.49-0.64)
	60-69 years	0.34 (0.30-0.39)	0.40 (0.31-0.53)	0.24 (0.20-0.28)	0.43 (0.36-0.52)
	70-79 years	0.45 (0.38-0.52)	0.53 (0.38-0.73)	0.39 (0.32-0.47)	0.55 (0.42-0.72)
	≥80 years	0.53 (0.45-0.62)	0.67 (0.51-0.90)	0.49 (0.41-0.59)	0.44 (0.32-0.61)
Death within 28 days after positive test	<10 years	--†	--†	--†	--†
	10-19 years	--†	--†	--†	--†
	20-29 years	--†	--†	--†	--†
	30-39 years	0.28 (0.07-1.04)	--†	0.20 (0.04-1.05)	0.21 (0.03-1.45)
	40-49 years	0.25 (0.11-0.55)	0.30 (0.07-1.24)	0.17 (0.07-0.44)	0.24 (0.06-1.02)
	50-59 years	0.16 (0.09-0.27)	0.57 (0.24-1.35)	0.11 (0.06-0.20)	0.17 (0.06-0.48)
	60-69 years	0.22 (0.15-0.34)	0.43 (0.20-0.92)	0.17 (0.10-0.27)	0.37 (0.15-0.91)
	70-79 years	0.26 (0.18-0.36)	0.30 (0.14-0.61)	0.23 (0.15-0.35)	0.34 (0.15-0.78)
	≥80 years	0.46 (0.36-0.58)	0.59 (0.37-0.93)	0.46 (0.34-0.60)	0.44 (0.26-0.75)

* Based on models stratified for calendar date, region, age group, ethnicity and vaccination status; and using regression adjustment for within-age-group linear age, sex, index of multiple deprivation and vaccine-status-specific reinfection status.
† Not estimated due to small numbers.

Table S8: Sensitivity analyses: effect of stratification versus regression adjustments for alternative or additional variables. Confounder adjustment by stratification is based on fewer model assumptions than adjustments using regression models. Stratification may however result in lower precision estimates due to a lower effective sample size, resulting from the omission of uninformative strata. The primary analysis used models stratified for calendar date, region, age group, ethnicity and vaccination status, and used regression to further adjust for within-age-group age differences, sex, index of multiple deprivation and vaccine status-specific reinfection status. To assess the sensitivity of the results to the set of variables used for stratification or regression adjustment, we (1) stratified for the narrower level upper tier local authority (UTLA) instead of region, (2) stratified for calendar date only and used regression adjustment for all other variables, (3) excluded one variable from the stratification set at a time and instead included it in the regression adjustment, and (4) additionally included one more variable in the stratification set at a time. All estimated HRs were consistent with those from the primary analysis, with the greatest variation being seen in the <10 age band. Stratification for UTLA instead of region or for additional covariates resulted in estimates with marginally wider CIs, and stratification for fewer covariates yielded estimates with marginally narrower CIs.

Outcome	Age group	HR (95% CI)*								
		Primary analysis	Alternative stratification							
			UTLA instead of region	Specimen date only	Not stratified for age group	Not stratified for region	Not stratified for ethnicity	Not stratified for vaccination status	Additional stratification for sex	Additional stratification for IMD
Hospital admission up to 14 days after positive test	<10 years	1.10 (0.85-1.42)	1.32 (0.94-1.87)	1.03 (0.89-1.20)	1.26 (1.08-1.47)	1.01 (0.80-1.28)	1.04 (0.82-1.33)	1.10 (0.85-1.43)	1.12 (0.85-1.46)	1.25 (0.92-1.70)
	10-19 years	0.83 (0.64-1.08)	0.81 (0.60-1.10)	0.81 (0.69-0.95)	0.96 (0.81-1.13)	0.78 (0.61-1.00)	0.79 (0.61-1.02)	0.91 (0.71-1.16)	0.84 (0.64-1.10)	0.89 (0.67-1.19)
	20-29 years	0.55 (0.48-0.63)	0.60 (0.50-0.71)	0.53 (0.48-0.58)	0.55 (0.49-0.60)	0.52 (0.45-0.59)	0.54 (0.47-0.62)	0.58 (0.51-0.67)	0.55 (0.48-0.64)	0.59 (0.51-0.70)
	30-39 years	0.44 (0.39-0.50)	0.43 (0.38-0.50)	0.44 (0.41-0.48)	0.45 (0.41-0.49)	0.44 (0.39-0.49)	0.44 (0.39-0.49)	0.46 (0.41-0.51)	0.44 (0.39-0.50)	0.44 (0.38-0.51)
	40-49 years	0.33 (0.29-0.38)	0.38 (0.31-0.45)	0.39 (0.36-0.43)	0.36 (0.33-0.40)	0.33 (0.29-0.37)	0.33 (0.28-0.37)	0.34 (0.30-0.39)	0.34 (0.29-0.39)	0.35 (0.30-0.41)
	50-59 years	0.26 (0.23-0.30)	0.31 (0.26-0.38)	0.32 (0.29-0.35)	0.28 (0.26-0.31)	0.26 (0.22-0.29)	0.25 (0.21-0.28)	0.25 (0.22-0.28)	0.26 (0.22-0.30)	0.28 (0.24-0.33)
	60-69 years	0.25 (0.21-0.30)	0.32 (0.24-0.42)	0.27 (0.24-0.30)	0.25 (0.22-0.27)	0.26 (0.22-0.30)	0.25 (0.22-0.29)	0.23 (0.19-0.26)	0.26 (0.22-0.32)	0.26 (0.21-0.33)
	70-79 years	0.36 (0.30-0.43)	0.31 (0.23-0.43)	0.27 (0.25-0.30)	0.27 (0.24-0.30)	0.37 (0.31-0.43)	0.37 (0.31-0.44)	0.32 (0.27-0.37)	0.37 (0.30-0.45)	0.33 (0.25-0.43)
	≥80 years	0.47 (0.40-0.56)	0.56 (0.40-0.77)	0.39 (0.35-0.43)	0.36 (0.33-0.40)	0.48 (0.42-0.56)	0.49 (0.42-0.58)	0.52 (0.45-0.60)	0.48 (0.40-0.59)	0.51 (0.39-0.67)
Any hospital attendance (including admission) up to 14 days after positive test	<10 years	1.03 (0.89-1.19)	1.10 (0.92-1.31)	0.92 (0.84-1.00)	1.14 (1.03-1.25)	1.00 (0.87-1.14)	1.00 (0.87-1.15)	1.03 (0.89-1.19)	1.05 (0.91-1.22)	1.04 (0.89-1.23)
	10-19 years	0.89 (0.76-1.03)	0.89 (0.75-1.06)	0.79 (0.72-0.87)	0.95 (0.86-1.05)	0.84 (0.73-0.98)	0.86 (0.75-1.00)	0.97 (0.84-1.12)	0.90 (0.77-1.04)	0.99 (0.84-1.16)
	20-29 years	0.67 (0.62-0.74)	0.70 (0.63-0.78)	0.65 (0.61-0.69)	0.67 (0.63-0.72)	0.65 (0.59-0.70)	0.67 (0.61-0.73)	0.71 (0.65-0.77)	0.68 (0.62-0.75)	0.69 (0.62-0.76)
	30-39 years	0.57 (0.52-0.61)	0.59 (0.54-0.64)	0.57 (0.54-0.60)	0.58 (0.54-0.61)	0.56 (0.52-0.60)	0.56 (0.52-0.61)	0.58 (0.54-0.63)	0.57 (0.52-0.61)	0.57 (0.52-0.62)
	40-49 years	0.54 (0.49-0.59)	0.59 (0.53-0.66)	0.58 (0.55-0.62)	0.54 (0.51-0.58)	0.53 (0.49-0.58)	0.53 (0.49-0.58)	0.55 (0.50-0.60)	0.54 (0.50-0.60)	0.56 (0.51-0.62)
	50-59 years	0.42 (0.38-0.46)	0.48 (0.42-0.55)	0.46 (0.43-0.49)	0.41 (0.38-0.44)	0.41 (0.37-0.45)	0.39 (0.35-0.43)	0.39 (0.35-0.43)	0.41 (0.37-0.46)	0.44 (0.39-0.50)
	60-69 years	0.32 (0.28-0.37)	0.41 (0.33-0.49)	0.34 (0.32-0.37)	0.32 (0.29-0.34)	0.32 (0.28-0.36)	0.32 (0.28-0.36)	0.27 (0.24-0.30)	0.34 (0.30-0.39)	0.35 (0.30-0.42)
	70-79 years	0.42 (0.36-0.50)	0.39 (0.30-0.50)	0.30 (0.28-0.33)	0.30 (0.27-0.32)	0.43 (0.38-0.50)	0.44 (0.38-0.50)	0.35 (0.30-0.40)	0.43 (0.36-0.51)	0.41 (0.33-0.51)
	≥80 years	0.49 (0.42-0.58)	0.60 (0.45-0.80)	0.40 (0.36-0.43)	0.38 (0.35-0.41)	0.50 (0.44-0.58)	0.53 (0.45-0.61)	0.49 (0.43-0.56)	0.50 (0.42-0.60)	0.52 (0.40-0.66)

Any hospital attendance (including admission) up to 14 days after positive test, or positive test during hospital stay	<10 years	1.04 (0.91-1.19)	1.07 (0.91-1.26)	0.93 (0.85-1.01)	1.14 (1.05-1.25)	1.03 (0.90-1.17)	1.02 (0.89-1.16)	1.04 (0.91-1.19)	1.05 (0.91-1.21)	1.04 (0.90-1.21)
	10-19 years	0.88 (0.77-1.00)	0.86 (0.74-1.00)	0.82 (0.75-0.89)	0.97 (0.89-1.06)	0.88 (0.77-1.00)	0.85 (0.75-0.97)	0.95 (0.84-1.08)	0.87 (0.76-1.00)	0.93 (0.81-1.08)
	20-29 years	0.70 (0.65-0.77)	0.73 (0.66-0.81)	0.69 (0.65-0.73)	0.71 (0.67-0.76)	0.68 (0.63-0.74)	0.70 (0.64-0.76)	0.74 (0.69-0.81)	0.72 (0.66-0.78)	0.72 (0.65-0.78)
	30-39 years	0.59 (0.55-0.63)	0.61 (0.56-0.66)	0.60 (0.57-0.63)	0.60 (0.57-0.64)	0.58 (0.54-0.62)	0.59 (0.55-0.63)	0.60 (0.56-0.65)	0.59 (0.55-0.63)	0.59 (0.55-0.64)
	40-49 years	0.57 (0.52-0.62)	0.64 (0.57-0.71)	0.63 (0.59-0.66)	0.58 (0.54-0.61)	0.57 (0.52-0.62)	0.57 (0.52-0.62)	0.58 (0.53-0.63)	0.58 (0.53-0.63)	0.60 (0.55-0.66)
	50-59 years	0.44 (0.40-0.49)	0.52 (0.46-0.59)	0.49 (0.46-0.52)	0.43 (0.41-0.46)	0.43 (0.39-0.47)	0.41 (0.38-0.45)	0.41 (0.38-0.45)	0.44 (0.40-0.49)	0.46 (0.41-0.52)
	60-69 years	0.34 (0.30-0.39)	0.44 (0.37-0.53)	0.36 (0.34-0.39)	0.33 (0.31-0.36)	0.34 (0.30-0.38)	0.34 (0.30-0.38)	0.29 (0.26-0.32)	0.36 (0.31-0.41)	0.38 (0.32-0.45)
	70-79 years	0.45 (0.38-0.52)	0.43 (0.33-0.55)	0.31 (0.29-0.34)	0.30 (0.28-0.33)	0.45 (0.39-0.52)	0.46 (0.40-0.52)	0.37 (0.32-0.42)	0.45 (0.38-0.54)	0.44 (0.35-0.54)
	≥80 years	0.53 (0.45-0.62)	0.60 (0.45-0.81)	0.39 (0.36-0.42)	0.37 (0.34-0.40)	0.53 (0.47-0.61)	0.56 (0.49-0.65)	0.53 (0.46-0.60)	0.54 (0.45-0.64)	0.55 (0.43-0.70)
Death within 28 days after positive test	<10 years	--†	--†	--†	--†	--†	--†	--†	--†	--†
	10-19 years	--†	--†	--†	--†	--†	--†	--†	--†	--†
	20-29 years	--†	--†	--†	--†	--†	--†	--†	--†	--†
	30-39 years	0.28 (0.07-1.04)	0.26 (0.05-1.19)	0.18 (0.09-0.37)	0.18 (0.09-0.37)	0.30 (0.09-1.00)	0.29 (0.08-1.03)	0.21 (0.06-0.74)	0.30 (0.08-1.16)	0.51 (0.11-2.25)
	40-49 years	0.25 (0.11-0.55)	0.27 (0.10-0.75)	0.37 (0.22-0.61)	0.32 (0.19-0.54)	0.25 (0.12-0.52)	0.27 (0.13-0.57)	0.21 (0.10-0.45)	0.27 (0.12-0.61)	0.22 (0.07-0.67)
	50-59 years	0.16 (0.09-0.27)	0.18 (0.09-0.37)	0.30 (0.22-0.40)	0.26 (0.18-0.35)	0.17 (0.11-0.28)	0.15 (0.09-0.24)	0.13 (0.08-0.21)	0.17 (0.10-0.29)	0.17 (0.09-0.32)
	60-69 years	0.22 (0.15-0.34)	0.20 (0.10-0.40)	0.27 (0.21-0.34)	0.26 (0.20-0.34)	0.23 (0.16-0.33)	0.21 (0.14-0.30)	0.17 (0.12-0.25)	0.22 (0.14-0.34)	0.16 (0.09-0.30)
	70-79 years	0.26 (0.18-0.36)	0.26 (0.14-0.47)	0.26 (0.21-0.31)	0.25 (0.20-0.31)	0.29 (0.22-0.39)	0.26 (0.19-0.36)	0.24 (0.18-0.32)	0.30 (0.20-0.43)	0.29 (0.17-0.50)
	≥80 years	0.46 (0.36-0.58)	0.50 (0.31-0.81)	0.42 (0.36-0.49)	0.41 (0.34-0.49)	0.50 (0.41-0.61)	0.50 (0.40-0.63)	0.56 (0.46-0.69)	0.46 (0.35-0.60)	0.46 (0.30-0.70)

* Based on models stratified for calendar date, region, age group, ethnicity and vaccination status, and using regression adjustment for within-age-group linear age, sex, index of multiple deprivation and vaccine-status-specific past infection status.

† Not estimated due to small numbers.

Table S9: Sensitivity analyses: effect of hospitalisation outcome definition. Primary analysis (which examined hospitalisation 0-14 days after positive test) compared with analysis where only hospitalisations 1-14 days after a positive test are included, or where the follow-up time is varied to instead include hospitalisations within 7, 28 or 60 days after a positive test.

Outcome	Age group	HR (95% CI)*				
		Primary analysis	Restricted to events 1 to 14 days after positive test	Events within 0 to 7 days after positive test	Events within 0 to 28 days after positive test	Events within 0 to 60 days after positive test
Hospital admission	<10 years	1.10 (0.85-1.42)	0.74 (0.50-1.09)	1.19 (0.90-1.58)	1.13 (0.90-1.41)	1.11 (0.90-1.38)
	10-19 years	0.83 (0.64-1.08)	0.83 (0.59-1.16)	0.76 (0.56-1.03)	0.91 (0.74-1.12)	0.91 (0.75-1.10)
	20-29 years	0.55 (0.48-0.63)	0.46 (0.39-0.55)	0.53 (0.45-0.62)	0.56 (0.50-0.63)	0.58 (0.51-0.65)
	30-39 years	0.44 (0.39-0.50)	0.41 (0.35-0.47)	0.40 (0.35-0.46)	0.49 (0.44-0.55)	0.50 (0.45-0.56)
	40-49 years	0.33 (0.29-0.38)	0.33 (0.28-0.39)	0.30 (0.25-0.35)	0.41 (0.36-0.46)	0.42 (0.37-0.47)
	50-59 years	0.26 (0.23-0.30)	0.35 (0.29-0.42)	0.23 (0.19-0.27)	0.30 (0.26-0.34)	0.32 (0.28-0.36)
	60-69 years	0.25 (0.21-0.30)	0.33 (0.26-0.42)	0.23 (0.19-0.28)	0.30 (0.26-0.35)	0.31 (0.26-0.36)
	70-79 years	0.36 (0.30-0.43)	0.50 (0.39-0.65)	0.32 (0.26-0.39)	0.39 (0.32-0.46)	0.39 (0.33-0.47)
	≥80 years	0.47 (0.40-0.56)	0.56 (0.43-0.74)	0.47 (0.39-0.56)	0.49 (0.41-0.58)	0.51 (0.43-0.60)
Any hospital attendance (including admission)	<10 years	1.03 (0.89-1.19)	0.96 (0.80-1.15)	1.06 (0.90-1.25)	1.03 (0.91-1.16)	1.01 (0.90-1.13)
	10-19 years	0.89 (0.76-1.03)	0.86 (0.72-1.03)	0.81 (0.67-0.98)	0.98 (0.88-1.09)	0.98 (0.89-1.08)
	20-29 years	0.67 (0.62-0.74)	0.65 (0.58-0.72)	0.63 (0.57-0.70)	0.75 (0.69-0.80)	0.78 (0.72-0.83)
	30-39 years	0.57 (0.52-0.61)	0.55 (0.51-0.60)	0.51 (0.47-0.56)	0.64 (0.60-0.68)	0.66 (0.62-0.70)
	40-49 years	0.54 (0.49-0.59)	0.55 (0.50-0.61)	0.46 (0.42-0.52)	0.64 (0.59-0.69)	0.66 (0.61-0.71)
	50-59 years	0.42 (0.38-0.46)	0.53 (0.46-0.60)	0.35 (0.31-0.40)	0.53 (0.48-0.58)	0.56 (0.51-0.61)
	60-69 years	0.32 (0.28-0.37)	0.41 (0.35-0.49)	0.29 (0.25-0.34)	0.41 (0.36-0.46)	0.43 (0.38-0.48)
	70-79 years	0.42 (0.36-0.50)	0.59 (0.48-0.73)	0.39 (0.33-0.46)	0.46 (0.40-0.53)	0.46 (0.40-0.53)
	≥80 years	0.49 (0.42-0.58)	0.55 (0.44-0.70)	0.49 (0.41-0.59)	0.55 (0.47-0.64)	0.57 (0.49-0.66)

* Based on models stratified for calendar date, region, age group, ethnicity and vaccination status; and using regression adjustment for within-age-group linear age, sex, index of multiple deprivation and vaccine-status-specific reinfection status.

Table S10: Sensitivity analyses: effect of epidemic phase bias. When two virus variants are in different phases of incidence growth or decline, controlling for the date of positive test may bias relative severity estimates if the mean time from infection to positive test is shorter for cases with more severe disease. Under this scenario, the apparent severity of the variant that is in a phase of growth may be overestimated and the severity of the variant with declining incidence may be underestimated. To examine the potential magnitude of this bias, a sensitivity analysis was recently proposed that applies a correction to the date of positive test for cases with the outcome of interest that corresponds to the assumed difference in mean time from infection to positive test between those without and with that outcome of interest.[1] We examined the effect of a difference of up to 2 days in the mean time from infection to positive test between cases who did not experience and cases who did experience each outcome, and refitted the primary analysis model stratified for the resulting proxy date. As expected, applying the correction resulted in somewhat lower adjusted HRs than in the primary analysis between cases with Omicron compared to Delta in all age groups.

**Outcome**	**Age group**	**HR (95% CI)***		
		**Assumed difference in mean time from infection to positive test between cases without outcome and cases with outcome**		
		**0 days (primary analysis)**	**1 day**	**2 days**
Hospital admission up to 14 days after positive test	<10 years	1.10 (0.85-1.42)	0.81 (0.62-1.04)	0.69 (0.53-0.89)
	10-19 years	0.83 (0.64-1.08)	0.60 (0.46-0.78)	0.46 (0.35-0.59)
	20-29 years	0.55 (0.48-0.63)	0.43 (0.38-0.50)	0.36 (0.31-0.41)
	30-39 years	0.44 (0.39-0.50)	0.35 (0.31-0.39)	0.27 (0.24-0.31)
	40-49 years	0.33 (0.29-0.38)	0.26 (0.23-0.30)	0.21 (0.18-0.24)
	50-59 years	0.26 (0.23-0.30)	0.21 (0.18-0.24)	0.17 (0.14-0.19)
	60-69 years	0.25 (0.21-0.30)	0.21 (0.18-0.25)	0.19 (0.16-0.22)
	70-79 years	0.36 (0.30-0.43)	0.30 (0.25-0.36)	0.26 (0.22-0.32)
	≥80 years	0.47 (0.40-0.56)	0.36 (0.30-0.43)	0.34 (0.29-0.41)
Any hospital attendance (including admission) up to 14 days after positive test	<10 years	1.03 (0.89-1.19)	0.78 (0.67-0.90)	0.63 (0.54-0.72)
	10-19 years	0.89 (0.76-1.03)	0.68 (0.58-0.79)	0.51 (0.44-0.59)
	20-29 years	0.67 (0.62-0.74)	0.53 (0.49-0.58)	0.44 (0.40-0.48)
	30-39 years	0.57 (0.52-0.61)	0.45 (0.42-0.48)	0.36 (0.33-0.38)
	40-49 years	0.54 (0.49-0.59)	0.43 (0.39-0.47)	0.34 (0.31-0.37)
	50-59 years	0.42 (0.38-0.46)	0.32 (0.29-0.36)	0.26 (0.23-0.29)
	60-69 years	0.32 (0.28-0.37)	0.25 (0.22-0.29)	0.22 (0.19-0.25)
	70-79 years	0.42 (0.36-0.50)	0.35 (0.30-0.41)	0.30 (0.26-0.35)
	≥80 years	0.49 (0.42-0.58)	0.39 (0.33-0.45)	0.36 (0.30-0.42)
Any hospital attendance (including admission) up to 14 days after positive test, or positive test during hospital stay	<10 years	1.04 (0.91-1.19)	0.79 (0.69-0.90)	0.63 (0.55-0.72)
	10-19 years	0.88 (0.77-1.00)	0.67 (0.59-0.77)	0.50 (0.44-0.57)
	20-29 years	0.70 (0.65-0.77)	0.55 (0.51-0.60)	0.46 (0.42-0.50)
	30-39 years	0.59 (0.55-0.63)	0.47 (0.43-0.50)	0.37 (0.35-0.40)
	40-49 years	0.57 (0.52-0.62)	0.45 (0.41-0.49)	0.36 (0.33-0.39)
	50-59 years	0.44 (0.40-0.49)	0.34 (0.31-0.38)	0.27 (0.25-0.30)
	60-69 years	0.34 (0.30-0.39)	0.27 (0.24-0.31)	0.24 (0.21-0.27)
	70-79 years	0.45 (0.38-0.52)	0.37 (0.32-0.43)	0.31 (0.27-0.36)
	≥80 years	0.53 (0.45-0.62)	0.41 (0.35-0.48)	0.38 (0.32-0.44)
Death within 28 days after positive test	<10 years	--†	--†	--†
	10-19 years	--†	--†	--†
	20-29 years	--†	--†	--†
	30-39 years	0.28 (0.07-1.04)	0.19 (0.05-0.77)	0.17 (0.04-0.70)
	40-49 years	0.25 (0.11-0.55)	0.20 (0.09-0.44)	0.16 (0.07-0.36)
	50-59 years	0.16 (0.09-0.27)	0.14 (0.08-0.24)	0.10 (0.06-0.18)
	60-69 years	0.22 (0.15-0.34)	0.17 (0.11-0.25)	0.14 (0.09-0.21)
	70-79 years	0.26 (0.18-0.36)	0.21 (0.15-0.31)	0.19 (0.13-0.26)
	≥80 years	0.46 (0.36-0.58)	0.37 (0.29-0.48)	0.31 (0.24-0.40)

* Based on models stratified for calendar date, region, age group, ethnicity and vaccination status; and using regression adjustment for within-age-group linear age, sex, index of multiple deprivation and vaccine-status-specific reinfection status.
† Not estimated due to small numbers.

Table S11. Sensitivity analysis: adjustment for under-ascertainment of past infections. Adjusted estimates for the unvaccinated population, from the secondary analysis, which estimated variant-specific HRs for hospitalisation or death in vaccinated groups versus unvaccinated, additionally to the Omicron vs Delta HRs, after imputation of undetected past infections. Undetected past infections in cases with no known past infections were imputed using the methods described in the appendix, p. xx. The HRs and 95% CIs were estimated using non-parametric bootstrapping (200 repetitions).

Outcome	Age group	HR (95% CI)*	
		Main analysis of intrinsic severity	Using imputation of under-ascertained past infections
Hospital admission within 14 days after positive test	<10 years	1.10 (0.85-1.42)	1.12 (0.86-1.44)
	10-19 years	0.78 (0.60-1.00)	0.87 (0.66-1.15)
	20-29 years	0.43 (0.37-0.49)	0.48 (0.42-0.55)
	30-39 years	0.31 (0.28-0.35)	0.35 (0.31-0.40)
	40-49 years	0.20 (0.17-0.23)	0.22 (0.19-0.25)
	50-59 years	0.14 (0.12-0.17)	0.16 (0.13-0.18)
	60-69 years	0.14 (0.12-0.16)	0.15 (0.13-0.18)
	70-79 years	0.20 (0.17-0.24)	0.21 (0.17-0.26)
	≥80 years	0.33 (0.28-0.39)	0.35 (0.28-0.41)
Any hospital attendance (including admission) within 14 days after positive test	<10 years	1.03 (0.89-1.19)	1.08 (0.94-1.24)
	10-19 years	0.84 (0.73-0.98)	0.92 (0.80-1.05)
	20-29 years	0.52 (0.48-0.57)	0.57 (0.51-0.63)
	30-39 years	0.40 (0.37-0.43)	0.43 (0.39-0.46)
	40-49 years	0.32 (0.29-0.35)	0.34 (0.31-0.38)
	50-59 years	0.22 (0.20-0.25)	0.24 (0.21-0.26)
	60-69 years	0.16 (0.14-0.18)	0.16 (0.14-0.19)
	70-79 years	0.19 (0.16-0.22)	0.20 (0.17-0.23)
	≥80 years	0.27 (0.23-0.31)	0.28 (0.23-0.32)
Any hospital attendance (including admission) within 14 days after positive test, or positive test during hospital admission	<10 years	1.04 (0.91-1.19)	1.08 (0.95-1.23)
	10-19 years	0.85 (0.75-0.97)	0.91 (0.81-1.02)
	20-29 years	0.55 (0.51-0.60)	0.59 (0.54-0.66)
	30-39 years	0.41 (0.38-0.45)	0.44 (0.40-0.48)
	40-49 years	0.34 (0.31-0.37)	0.36 (0.33-0.40)
	50-59 years	0.24 (0.22-0.26)	0.25 (0.23-0.28)
	60-69 years	0.17 (0.15-0.19)	0.18 (0.16-0.20)
	70-79 years	0.20 (0.18-0.24)	0.21 (0.18-0.25)
	≥80 years	0.29 (0.25-0.34)	0.30 (0.26-0.34)
Death within 28 days after positive test	<10 years	--§	--§
	10-19 years	--§	--§
	20-29 years	--§	--§
	30-39 years	0.13 (0.04-0.46)	0.16 (0.04-0.63)
	40-49 years	0.11 (0.05-0.24)	0.13 (0.06-0.26)
	50-59 years	0.07 (0.04-0.12)	0.08 (0.05-0.14)
	60-69 years	0.11 (0.07-0.16)	0.11 (0.07-0.18)
	70-79 years	0.16 (0.11-0.22)	0.16 (0.11-0.23)
	≥80 years	0.36 (0.27-0.48)	0.37 (0.27-0.51)

Table S12. Sensitivity analysis: adjustment for under-ascertainment of past infections. HR estimates for vaccination and prior infection categories for the model used to estimate age-specific hazard ratios (HRs) for Omicron versus Delta and variant-specific vaccine effectiveness. Corresponding age-specific hazard ratios for Omicron vs Delta cases in unvaccinated individuals are shown in the rightmost column of Table 1. HRs for vaccination categories are versus unvaccinated cases, those for past infection categories are versus cases with no documented past infection. Vaccination categories are stratified by vaccine given for doses 1 and 2, number of doses received (dose 3 was always Pfizer or Moderna booster), and weeks elapsed from last dose to positive SARS-CoV-2 specimen date.

Variable	HR (95% CI)			
	Hospital admission up to 14 days after positive test	Any hospital attendance (including admission) up to 14 days after positive test	Any hospital attendance (including admission) up to 14 days after positive test, or positive test during hospital stay	Death within 28 days after positive test
Delta: AstraZeneca, dose 1, any time*	0.55 (0.45-0.65)	0.65 (0.57-0.75)	0.69 (0.60-0.78)	0.53 (0.33-0.90)
Delta: AstraZeneca, dose 2, <2 weeks	0.16 (0.06-0.33)	0.23 (0.11-0.42)	0.23 (0.11-0.41)	–
Delta: AstraZeneca, dose 2, 2-7 weeks	0.19 (0.00-0.53)	0.22 (0.06-0.46)	0.22 (0.06-0.45)	–
Delta: AstraZeneca, dose 2, 8-11 weeks	0.59 (0.17-1.39)	0.50 (0.20-0.96)	0.49 (0.20-0.95)	–
Delta: AstraZeneca, dose 2, 12-15 weeks	0.32 (0.14-0.54)	0.31 (0.17-0.48)	0.32 (0.19-0.49)	–
Delta: AstraZeneca, dose 2, 16-19 weeks	0.14 (0.09-0.19)	0.23 (0.18-0.29)	0.26 (0.21-0.31)	–
Delta: AstraZeneca, dose 2, 20+ weeks	0.21 (0.19-0.23)	0.27 (0.26-0.29)	0.29 (0.28-0.31)	0.26 (0.21-0.31)
Delta: AstraZeneca, dose 3, <2 weeks	0.10 (0.09-0.11)	0.15 (0.13-0.16)	0.16 (0.15-0.18)	0.10 (0.07-0.15)
Delta: AstraZeneca, dose 3, 2-7 weeks	0.13 (0.11-0.15)	0.17 (0.15-0.19)	0.18 (0.16-0.21)	0.12 (0.08-0.16)
Delta: AstraZeneca, dose 3, 8-11 weeks	0.14 (0.11-0.18)	0.21 (0.18-0.26)	0.23 (0.19-0.27)	0.14 (0.10-0.20)
Delta: AstraZeneca, dose 3, 12+ weeks	0.28 (0.14-0.45)	0.32 (0.18-0.57)	0.34 (0.18-0.58)	–
Delta: Pfizer/Moderna, dose 1, <2 weeks	0.56 (0.37-0.76)	0.68 (0.54-0.83)	0.71 (0.57-0.84)	–
Delta: Pfizer/Moderna, dose 1, 2-7 weeks	0.33 (0.23-0.44)	0.45 (0.37-0.54)	0.49 (0.40-0.57)	0.64 (0.17-1.54)
Delta: Pfizer/Moderna, dose 1, 8-11 weeks	0.39 (0.22-0.54)	0.46 (0.36-0.57)	0.56 (0.46-0.69)	–
Delta: Pfizer/Moderna, dose 1, 12+ weeks	0.36 (0.30-0.42)	0.43 (0.38-0.48)	0.47 (0.41-0.52)	0.36 (0.17-0.66)
Delta: Pfizer/Moderna, dose 2, <2 weeks	0.18 (0.06-0.36)	0.33 (0.21-0.46)	0.32 (0.21-0.45)	–
Delta: Pfizer/Moderna, dose 2, 2-7 weeks	0.43 (0.25-0.65)	0.40 (0.29-0.56)	0.46 (0.34-0.63)	–
Delta: Pfizer/Moderna, dose 2, 8-11 weeks	0.24 (0.14-0.34)	0.31 (0.23-0.38)	0.34 (0.26-0.43)	–
Delta: Pfizer/Moderna, dose 2, 12-15 weeks	0.14 (0.10-0.19)	0.21 (0.18-0.25)	0.24 (0.21-0.28)	–
Delta: Pfizer/Moderna, dose 2, 16-19 weeks	0.10 (0.08-0.13)	0.18 (0.16-0.20)	0.20 (0.18-0.22)	–
Delta: Pfizer/Moderna, dose 2, 20+ weeks	0.18 (0.17-0.21)	0.25 (0.23-0.27)	0.27 (0.25-0.29)	0.28 (0.22-0.36)
Delta: Pfizer/Moderna, dose 3, <2 weeks	0.13 (0.10-0.17)	0.17 (0.14-0.20)	0.18 (0.15-0.21)	0.14 (0.09-0.21)
Delta: Pfizer/Moderna, dose 3, 2-7 weeks	0.12 (0.10-0.14)	0.18 (0.16-0.21)	0.19 (0.17-0.22)	0.13 (0.09-0.18)
Delta: Pfizer/Moderna, dose 3, 8-11 weeks	0.11 (0.09-0.13)	0.16 (0.14-0.18)	0.17 (0.15-0.19)	0.10 (0.07-0.13)
Delta: Pfizer/Moderna, dose 3, 12+ weeks	0.15 (0.10-0.22)	0.19 (0.13-0.25)	0.19 (0.13-0.26)	0.11 (0.04-0.21)
Omicron: AstraZeneca, dose 1, any time*	1.09 (0.91-1.27)	1.17 (1.03-1.30)	1.17 (1.03-1.30)	0.96 (0.62-1.64)
Omicron: AstraZeneca, dose 2, <2 weeks	0.56 (0.28-0.96)	0.61 (0.38-0.86)	0.57 (0.35-0.81)	–
Omicron: AstraZeneca, dose 2, 2-7 weeks	0.34 (0.18-0.55)	0.48 (0.32-0.62)	0.46 (0.32-0.61)	–
Omicron: AstraZeneca, dose 2, 8-11 weeks	0.59 (0.32-0.92)	0.63 (0.40-0.90)	0.67 (0.40-0.89)	0.69 (0.26-1.77)
Omicron: AstraZeneca, dose 2, 12-15 weeks	0.44 (0.24-0.76)	0.54 (0.35-0.76)	0.57 (0.39-0.80)	–
Omicron: AstraZeneca, dose 2, 16-19 weeks	0.48 (0.33-0.67)	0.59 (0.46-0.74)	0.63 (0.48-0.78)	–
Omicron: AstraZeneca, dose 2, 20+ weeks	0.50 (0.46-0.54)	0.56 (0.53-0.60)	0.60 (0.56-0.63)	0.79 (0.62-1.06)
Omicron: AstraZeneca, dose 3, <2 weeks	0.26 (0.22-0.29)	0.34 (0.31-0.37)	0.37 (0.34-0.39)	0.25 (0.15-0.41)
Omicron: AstraZeneca, dose 3, 2-7 weeks	0.20 (0.18-0.22)	0.31 (0.30-0.34)	0.34 (0.32-0.36)	0.16 (0.12-0.23)
Omicron: AstraZeneca, dose 3, 8-11 weeks	0.19 (0.17-0.21)	0.39 (0.36-0.42)	0.40 (0.37-0.44)	0.14 (0.11-0.20)
Omicron: AstraZeneca, dose 3, 12+ weeks	0.21 (0.17-0.25)	0.44 (0.38-0.50)	0.45 (0.39-0.51)	0.21 (0.14-0.31)
Omicron: Pfizer/Moderna, dose 1, <2 weeks	0.61 (0.44-0.82)	0.68 (0.56-0.80)	0.67 (0.58-0.80)	–
Omicron: Pfizer/Moderna, dose 1, 2-7 weeks	0.42 (0.33-0.51)	0.55 (0.48-0.61)	0.55 (0.48-0.61)	–
Omicron: Pfizer/Moderna, dose 1, 8-11 weeks	0.45 (0.33-0.61)	0.53 (0.44-0.62)	0.55 (0.47-0.63)	0.55 (0.10-1.77)
Omicron: Pfizer/Moderna, dose 1, 12+ weeks	0.58 (0.48-0.66)	0.66 (0.58-0.73)	0.69 (0.62-0.75)	0.51 (0.20-0.99)
Omicron: Pfizer/Moderna, dose 2, <2 weeks	0.48 (0.34-0.62)	0.55 (0.44-0.65)	0.54 (0.45-0.63)	–
Omicron: Pfizer/Moderna, dose 2, 2-7 weeks	0.35 (0.27-0.45)	0.44 (0.37-0.51)	0.48 (0.41-0.55)	0.94 (0.26-2.53)
Omicron: Pfizer/Moderna, dose 2, 8-11 weeks	0.34 (0.26-0.41)	0.48 (0.42-0.55)	0.50 (0.45-0.56)	–
Omicron: Pfizer/Moderna, dose 2, 12-15 weeks	0.28 (0.24-0.33)	0.35 (0.32-0.38)	0.38 (0.35-0.41)	–
Omicron: Pfizer/Moderna, dose 2, 16-19 weeks	0.22 (0.19-0.25)	0.31 (0.29-0.34)	0.35 (0.32-0.37)	–

Omicron: Pfizer/Moderna, dose 2, 20+ weeks	0.38 (0.34-0.42)	0.48 (0.45-0.51)	0.50 (0.47-0.53)	0.73 (0.53-0.98)
Omicron: Pfizer/Moderna, dose 3, <2 weeks	0.20 (0.18-0.23)	0.29 (0.27-0.32)	0.33 (0.30-0.35)	0.24 (0.11-0.41)
Omicron: Pfizer/Moderna, dose 3, 2-7 weeks	0.23 (0.20-0.26)	0.39 (0.36-0.42)	0.41 (0.38-0.45)	0.19 (0.13-0.27)
Omicron: Pfizer/Moderna, dose 3, 8-11 weeks	0.19 (0.18-0.22)	0.37 (0.35-0.40)	0.39 (0.36-0.42)	0.13 (0.10-0.18)
Omicron: Pfizer/Moderna, dose 3, 12+ weeks	0.17 (0.15-0.19)	0.35 (0.32-0.38)	0.36 (0.33-0.40)	0.11 (0.08-0.16)
Past infection vs no past infection, unvaccinated	0.46 (0.38-0.53)	0.63 (0.56-0.71)	0.68 (0.60-0.75)	0.10 (0.00-0.22)
Past infection vs no past infection, vaccinated	0.85 (0.75-0.96)	1.02 (0.96-1.09)	1.02 (0.97-1.09)	0.31 (0.19-0.44)

* Due to small numbers, all cases who had received a single dose of the AstraZeneca vaccine are grouped together.

Supplementary methods. Sensitivity analysis adjusting for under-ascertainment of past infections

The secondary analysis estimated intrinsic severity in unvaccinated cases based on a model that included vaccination-status-specific effects of known past infections. To account for the possibility that a fraction of those with no known past infections have had an untested past infection, we imputed past infections in a sensitivity analysis. The imputations for cases with no observed past infections were based on the model

$$\Pr(r' = 1|X,\ r = 0) = \frac{\Pr(r = 1|X)/\Pr(r = 0|X)}{\Pr(r = 1|X,\ r' = 1)/\Pr(r = 0|X,\ r' = 1)}$$

where r and r' are indicator variables for whether a case has an observed and a true past infection, respectively, and X are other individual-level covariates. This equality follows from Bayes' theorem, noting that $\Pr(r = 1|X) = \Pr(r = 1|X,\ r' = 1)\ \Pr(r' = 1|X)$ under the assumption that $\Pr(r = 1|X,\ r' = 0) = 0$. No imputation was done for the past infection status of cases with observed past infections. We estimated the numerator based on the observed frequencies of cases with observed past infection or no past infection in strata defined by age group, date of positive test, vaccination status, variant, and outcome.

For the denominator, we used estimates of the mean cumulative population infection incidence up to 28 November 2021, based on an independent model of age-stratified population infection prevalence (https://github.com/epiforecasts/inc2prev/) that used data from the ONS infection survey[2] and from UKHSA serological surveys[3].

We assumed that the number of infections per person in age group a up to 28 November 2021, I_a, follow a Poisson distribution parametrised by the estimated mean cumulative per-capita infection incidence, m_a. We then estimated, for cases after 28 November 2021, the probability that a previous infection would have been observed as $\Pr(r = 1|a,\ r' = 1) \approx (n_a/N_a)/\Pr(I_a \geq 1)$. Here n_a is the number of unique individuals in age group a who had tested positive for SARS-CoV-2 up to 28 November 2021 (from the national line list maintained by UKHSA[4]), N_a is the population size of age group a (mid-2020 estimates; https://www.ons.gov.uk/peoplepopulationandcommunity/populationandmigration/populationestimates/datasets/populationestimatesforukenglandandwalesscotlandandnorthernireland), and $\Pr(I_a \geq 1) = 1 - \exp(-m_a)$ is the Poisson probability that individuals in age group a had been infected at least once up to 28 November 2021. The table below summarises the resulting estimates.

Age band	A. Estimated mean cumulative infections per person up to 28/11/2021	B. Standard deviation of posterior of cumulative infection incidence per person	C. Proportion of people with 1 or more past infections = 1-exp(column A)	D..Number of people with one or more infections detected up to 28/11/2021	E. Population size (ONS)	F. Proportion of population who have had one or more infections detected = column D /column E	G. Proportion of all past first infections reported =column F /column C
2-10	0.92	0.033	0.60	582781	6254603	0.09	0.16
11-15	1.21	0.046	0.70	853211	3370248	0.25	0.36
16-24	0.87	0.029	0.58	1344870	5950637	0.23	0.39
25-34	0.56	0.020	0.43	1463940	7596145	0.19	0.45
35-49	0.57	0.023	0.44	1977072	10853151	0.18	0.42
50-69	0.42	0.015	0.34	1741162	13618246	0.13	0.37
70+	0.27	0.010	0.24	619572	7679719	0.08	0.34

We used linear interpolation (using the mid-point of age bands) to estimate $\Pr(r = 1|a,\ r' = 1)$ and its posterior standard deviation for <1, 1-4, 5-9 and then 10 year age bands from the estimates with the age stratification given in the above table, making the assumption that the values for <1s and 1-4 year-olds were the same as those estimated for 2-10 year-olds above.

To account for the added uncertainty, used a non-parametric bootstrap (200 repetitions) of all cases in the study period. For each repetition, we first sampled $\Pr(r = 1|a,\ r' = 1)$ from the posterior distribution for each age band, and then used the above imputation model to sample "true" reinfection status for every case with no known past infection. We then refitted the secondary analysis model (that estimates intrinsic severity and the effect of vaccine status and past infection) to the resulting imputed dataset. We estimated HRs from the bootstrap medians and 95% CIs based on the bootstrap quantiles.

References

1. Seaman SR, Nyberg T, Overton CE, Pascall D, Presanis AM, De Angelis D. Adjusting for time of infection or positive test when estimating the risk of a post-infection outcome in an epidemic. *medRxiv* 2021: 2021.08.13.21262014.
2. Vöhringer HS, Sanderson T, Sinnott M, et al. Genomic reconstruction of the SARS-CoV-2 epidemic in England. *Nature* 2021; **600**(7889): 506-11.
3. UK Health Security Agency. COVID-19 vaccine surveillance report: Week 6. 2022. https://www.gov.uk/government/publications/covid-19-vaccine-weekly-surveillance-reports.
4. Clare T, Twohig KA, O'Connell AM, Dabrera G. Timeliness and completeness of laboratory-based surveillance of COVID-19 cases in England. *Public Health* 2021; **194**: 163-6.
